# *Giardia duodenalis*: Flavohemoglobin is involved in drug biotransformation and resistance to albendazole

**DOI:** 10.1371/journal.ppat.1010840

**Published:** 2022-09-27

**Authors:** Edar O. Pech-Santiago, Raúl Argüello-García, Citlali Vázquez, Emma Saavedra, Iliana González-Hernández, Helgi Jung-Cook, Steven P. Rafferty, M. Guadalupe Ortega-Pierres

**Affiliations:** 1 Departamento de Genética y Biología Molecular, Centro de Investigación y de Estudios Avanzados del Instituto Politécnico Nacional, Mexico City, Mexico; 2 Departamento de Bioquímica, Instituto Nacional de Cardiología Ignacio Chávez, Mexico City, Mexico; 3 Laboratorio de Neuropsicofarmacología, Instituto Nacional de Neurología y Neurocirugía, Manuel Velasco Suárez, Ciudad de México, México; 4 Department of Chemistry, Trent University, Peterborough, Canada; Heidelberg University, GERMANY

## Abstract

*Giardia duodenalis* causes giardiasis, a major diarrheal disease in humans worldwide whose treatment relies mainly on metronidazole (MTZ) and albendazole (ABZ). The emergence of ABZ resistance in this parasite has prompted studies to elucidate the molecular mechanisms underlying this phenomenon. *G*. *duodenalis* trophozoites convert ABZ into its sulfoxide (ABZSO) and sulfone (ABZSOO) forms, despite lacking canonical enzymes involved in these processes, such as cytochrome P450s (CYP450s) and flavin-containing monooxygenases (FMOs). This study aims to identify the enzyme responsible for ABZ metabolism and its role in ABZ resistance in *G*. *duodenalis*. We first determined that the iron-containing cofactor heme induces higher mRNA expression levels of flavohemoglobin (gFlHb) in *Giardia* trophozoites. Molecular docking analyses predict favorable interactions of gFlHb with ABZ, ABZSO and ABZSOO. Spectral analyses of recombinant gFlHb in the presence of ABZ, ABZSO and ABZSOO showed high affinities for each of these compounds with *K*_*d*_ values of 22.7, 19.1 and 23.8 nM respectively. ABZ and ABZSO enhanced gFlHb NADH oxidase activity (turnover number 14.5 min^-1^), whereas LC-MS/MS analyses of the reaction products showed that gFlHb slowly oxygenates ABZ into ABZSO at a much lower rate (turnover number 0.01 min^-1^). Further spectroscopic analyses showed that ABZ is indirectly oxidized to ABZSO by superoxide generated from the NADH oxidase activity of gFlHb. In a similar manner, the superoxide-generating enzyme xanthine oxidase was able to produce ABZSO in the presence of xanthine and ABZ. Interestingly, we find that gFlHb mRNA expression is lower in albendazole-resistant clones compared to those that are sensitive to this drug. Furthermore, all albendazole-resistant clones transfected to overexpress gFlHb displayed higher susceptibility to the drug than the parent clones. Collectively these findings indicate a role for gFlHb in ABZ conversion to its sulfoxide and that gFlHb down-regulation acts as a passive pharmacokinetic mechanism of resistance in this parasite.

## Author summary

A major concern in the treatment of infectious diseases is the increased prevalence and distribution of drug resistance in microorganisms. In relation to giardiasis, there is an increase resistance to widespread, routinely used drugs, including albendazole (ABZ). Although *G*. *duodenalis* may convert ABZ to its mono-oxygenated form, albendazole sulfoxide (ABZSO), and further oxidize it to the less active albendazole sulfone (ABZSOO), the canonical molecular machineries for such transformations, namely cytochrome P450s (CYP450s) and flavin monooxygenases (FMOs), are not encoded in the genome of this parasite. In this work, we show that *G*. *duodenalis* flavohemoglobin (gFlHb) contributes to ABZ → ABZSO biotransformation at least via oxygenation, mediated by superoxide derived from its NADH oxidase activity, and that the mRNA expression of the respective gene is down-regulated in ABZ-resistant parasites, suggesting that this enzyme is inversely related to the ABZ-resistant phenotype. Consequently, gFlHb seems to play a novel function/mechanism in ABZ biotransformation, reinforcing its status as a novel therapeutic target against *G*. *duodenalis*, particulrly since it is absent from the human host.

## Introduction

*Giardia duodenalis* (syn. *G*. *lamblia*, *G*. *intestinalis*) is an intestinal protozoan parasite and the causative agent of giardiasis, an intestinal disease affecting humans and other mammals [1,2]⁠. Since 2004, giardiasis has been included in the Neglected Diseases Initiative of the World Health Organization (WHO) [3]⁠. The control of giardiasis mainly relies in the use of drugs. Among the most common drugs used for the treatment of giardiasis are metronidazole (MTZ) and albendazole (ABZ); the latter has fewer side effects, and its effectiveness on the parasite is similar to that observed with MTZ [4,5].

**Fig 1:**
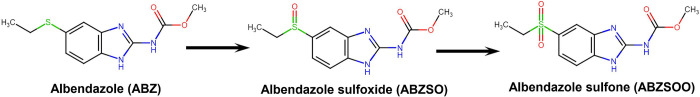
Chemical structure of ABZ, ABZSO, ABZSOO and schematic pathway of ABZ metabolism.

ABZ is a broad-spectrum parasiticide that binds to monomeric β-tubulin and heterodimeric αβ-tubulin preventing its polymerization; this in turn destabilizes microtubules within the cell, thus inhibiting mobility and molecular transport [6,7]⁠. In higher eukaryotes, ABZ is transformed to sulfoxide (ABZSO), the principal cytotoxic metabolite, and subsequently into sulfone (ABZSOO) through the action mainly of the cytochrome P450 monooxygenases (CYPs) and by flavin-containing monooxygenase (FMO) (Fig 1) [8,9]⁠. CYPs are heme proteins that catalyze the biotransformation of a wide variety of chemically and structurally diverse compounds, including > 85% of currently used therapeutic drugs. CYPs activate molecular oxygen by the sequential input of two electrons through the activities of NADPH-cytochrome P450 oxidoreductase (CPR) and cytochrome *b*_5_ (cyt *b*_5_) [10]⁠. Interestingly, *Giardia* carries out ABZ biotransformation into ABZSO and ABZSOO, despite the absence of both FMO and CYP [11]⁠. Intriguingly, *Giardia* does possess two members of the CPR family (mitosomal GiOR-1, GL50803_91252; non-mitosomal GiOR-2, GL50803_15897) [12]⁠ and four members of the cyt *b*_5_ family [13,14].⁠

The clinical efficacy of anti-giardial drugs is crucial for the control of *Giardia* infections but therapeutic failures have been reported [15–18]⁠; these failures could be due to treatment noncompliance, use of suboptimal doses of the drug and/or the use of single doses treatments in parasite control campaigns [18,19]⁠. Resistance to various drugs has been induced *in vitro* in *G*. *duodenalis* trophozoites, and some field isolates with resistance to MTZ have been identified. In particular, the molecular basis of ABZ resistance in this parasite has not yet been fully elucidated. In helminths, resistance to ABZ has been associated with single-nucleotide polymorphisms in three codons (167, 198, and 200) of the β-tubulin gene, which change the β-tubulin structure of the protein and alter its binding to ABZ [20–23]⁠. However, in ABZ-resistant clones of *Giardia* trophozoites a different mutation (E198K) in β-tubulin was recently discovered, suggesting that ABZ-resistance in *Giardia* may have different mechanisms related to the pharmacokinetics or pharmacodynamics of the compound [18,24,25]⁠. In this context, our previous studies of ABZ resistance in this parasite showed that resistant trophozoites accumulated lower levels of ABZ metabolites, had increased mRNA expression of genes that encode enzymes involved in detoxification of reactive oxygen species (NADH oxidase, peroxiredoxin 1a and flavodiiron protein), a so-called ABZ resistance-related variant surface protein (ARR-VSP) and an increased thiol pool [11,26,27]⁠. These changes might be associated with an adaptive fitness cost as observed for MTZ-resistant parasites [28].⁠

Although *Giardia* lacks mitochondria and is not able to synthesize heme [29]⁠, it has at least five heme proteins; four of them are members of the cytochrome *b*_5_ family of electron transfer proteins [13,14]⁠ and one enzyme belongs to the flavohemoglobin class [30]. Flavohemoglobins (FlHbs) possess a C-terminal domain homologous to ferredoxin:NADPH reductase, with highly conserved binding sites for FAD and NAD(P)H, and a N-terminal heme-binding microbial globin domain [31,32]⁠. Flavohemoglobins are best known for their ability to act as nitric oxide dioxygenases (NODs), in which an electron supplied by a reduced nicotinamide cofactor is transferred via FAD to the globin domain where it combines with molecular oxygen and nitric oxide (NO) to produce nitrate. In this way the potentially damaging free radical NO is consumed. Consistent with this role, the levels of gFlHb mRNA, protein, and NOD activity are all increased in *Giardia* trophozoites subjected to nitrosative stress in culture [33]⁠. The structures of FlHbs reveal marked plasticity and a flexible distal pocket that can accommodate large molecules including phospholipids, suggesting that this protein has additional function(s) [34,35]⁠. Indeed, flavohemoglobins also possess alkylhydroperoxide reductase and NO reductase activities [36,37]⁠ in addition to their NOD activity.

In this study, we explored the role of gFlHb in ABZ biotransformation and ABZ resistance in *Giardia* trophozoites. We hypothesized that gFlHb promotes the conversion of ABZ to ABZSO which is likely linked to an antioxidant, pharmacokinetic mechanism of resistance to ABZ in *G*. *duodenalis*.

## Material and methods

### Chemicals and biological reagents

ABZ, ABZSO, ABZSOO and carbamazepine (CBZ, internal standard) were purchased from Sigma-Aldrich Co. (St Louis, MO, USA). Methanol, acetonitrile, ether, dichloromethane, and chloroform were of HPLC grade (Mallinckrodt Co., St. Louis, MO, USA). Formic acid (Sigma-Aldrich Co.) was of analytical reagent-grade. Water was obtained from a Milli-Q Water System (Millipore Corporation, Bedford, MA, USA).

### Parasite cultures: ABZ-susceptible and ABZ-resistant clones

*G*. *duodenalis* (WB strain, Assemblage A, ATCC # 30957) were grown at 37°C in 4.5 mL screw capped vials in Keister´s modified TYI-S-33 medium (Keister, 1983) containing 10% bovine serum (Hyclone, South Logan, UT, USA) with 1% antibiotic/antimycotic mixture (HyClone, South Logan, UT, USA). The ABZ-resistant clones able to grow under 1.35 (R1.35), 8.0 (R8.0), and 250 (R250) μM concentrations of ABZ respectively, were obtained as previously described [18]⁠. A control culture of an ABZ-susceptible clone grown in the presence of 0.5% v/v dimethylformamide (WB-DMF), was used throughout this study. Trophozoites were harvested by chilling the tubes in an iced water bath for 1 h. Detached trophozoites were centrifuged at 750 x *g* for 10 min at 4°C, the cell pellet was washed three times with PBS and parasite viability was determined by the Trypan blue exclusion assay. For this purpose, the pelleted cells were suspended in 1mL PBS and one part of Trypan blue (0.8%) and one part of cell suspension were mixed and incubated for 5 min at room temperature. Viable (non-stained) and non-viable (stained) cells were counted using a Neubauer hemocytometer.

### Hemin susceptibility assays

Cultures were grown in culture medium supplemented with increasing hemin concentrations (4–250 μM) (Sigma-Aldrich Co.) and incubated at 37°C for 24 h. At the end of incubation, trophozoite viability was determined by Trypan blue exclusion as described above.

### Molecular modeling and docking

The amino acid sequence of gFlHb was obtained from the sequence of GL50803_15009 deposited in the GiardiaDB [38]⁠. Sequence alignments were performed using the Clustal Omega program server (https://www.ebi.ac.uk/Tools/msa/clustalo/) [39]⁠. The three-dimensional model was built using the I-TASSER webserver (https://zhanglab.ccmb.med.umich.edu/I-TASSER/) [40]⁠. The models were evaluated by PROCHECK [41] and VERYFY 3D [42,43]⁠ of the SAVE v5.0 platform (https://servicesn.mbi.ucla.edu/SAVES/), and ProSA-Web [44]⁠ (https://prosa.services.came.sbg.ac.at/prosa.php). Predicted secondary structures of modelled proteins were determined using PSIPRED 4.0 [45] ⁠https://prosa.services.came.sbg.ac.at/prosa.php). Structural alignment of gFlHb with Hmp (PDB: 1GVH) was carried out with TM-ALIGN [46]⁠).

Chemical structures of ABZ (ZINC17146904), ABZSO (ZINC4095934) and AZBSOO (ZINC5424275), and the azole inhibitors econazole (ZINC596881), ketoconazole (ZINC643153) and miconazole (ZINC607971) were retrieved from the ZINC database [47]⁠. The protein-ligand docking studies were performed using SwissDock [48]⁠ and AutoDock Vina [49]⁠ tool of the UCSF-Chimera v 1.13.1 software. Initially, the active pocket was identified with known structures of flavohemoglobins of *E*. *coli* (Hmp: 1GVH), *Alcaligenes eutropha* (AeFlHb: 1CQX) and yeast (YHB1: 4G1V). Subsequently, the heme axial ligand HisF8 was localized at the center with a grid box of 20 x 20 x 20 points and the drugs compounds were docked with gFlHb and Hmp models. The best binding mode of each molecule was selected based on the lowest binding free energy (kcal/mol) and root-mean-square deviation (rmsd).

### Cloning, expression, and purification of gFlHb

*E*. *coli* strain BL21 (DE3) was transformed using the *Giardia* flavohemoglobin expression vector pET14b-H6-gFlHb which encodes gFlHb fused to an N-terminal His6-tag and confers resistance to ampicillin [30]⁠. One-liter Fernbach flasks containing 400 mL of Terrific Broth supplemented with 50 μg/ mL ampicillin were inoculated with 5 mL of overnight starter cultures of transformed cells and incubated with shaking for 24 hours at 37°C. Cells from each flask were harvested by centrifugation at 5000 x *g* for 10 min at 4°C and resuspended in 15 mL binding buffer (0.05 M Tris-HCl, 0.5 M NaCl, pH 7.5) supplemented with a protease inhibitor cocktail (Complete, Roche). The cell suspension was placed on ice and processed by sonication. Purification of recombinant gFlHb using nickel affinity column was performed as described in Rafferty *et al*. (2010) [30]⁠. The purified protein was stored at -20°C in glycerol at 50% final concentration. Protein concentration was determined using a Pierce BCA Protein Assay Kit (Thermo Scientific) and the absorbance was measured at 562 nm in a microplate reader (BioRad). Protein concentration was also determined in samples of purified protein precipitated with trichloroacetic acid to eliminate imidazole and later resuspended in Lowry´s solution A (Na_2_CO_3_, 0.4% NaOH, 0.16% sodium-potassium tartrate, 1% SDS).

### Spectroscopic characterization and enzyme activity of gFlHb

UV-visible absorption spectra of ferric and NADH-reduced (ferrous oxygenated) gFlHb were recorded on a Cary 60 Bio UV-VIS spectrophotometer (Agilent; Santa Clara, CA, USA) using quartz cuvettes of 1 cm path. Formation of the ABZ complex was assessed by the change of the high spin form of the protein upon the addition of ABZ using visible absorption spectroscopy. For this, measurements of the absorption difference between the ligand-free maxima (415 nm) and the fully ligand-bound (425 nm) heme were followed at 37°C, using a solution of 6.6 μM gFlHb in air-equilibrated 50 mM sodium phosphate buffer at pH 7.5, 150 μM NADH, in a total volume of 1 mL. DMF was present in the cuvette at a final concentration of less than 1% (*v/v*). UV-visible absorption spectra of gFlHb under anaerobic conditions were recorded in a capped quartz cuvette equipped with a pierceable rubber septum, and the solution was deoxygenated with a stream of argon for 30 minutes. The absorption spectrum measurements were determined in a mixture of 2.4 mL of 0.1 M Bis-Tris buffer at pH 6.5 and 100 μL of 16.3 μM of gFlHb to obtain a concentration of 0.65 μM in enzyme. The ferrous deoxygenated state was generated in this sample by addition of 30 μL of freshly-prepared sodium dithionite in deoxygenated water; finally, 2.5 μL of 5 mM ABZ in DMSO was added for a final concentration of 5 μM ABZ.

The dissociation constants (*K*_*d*_) of gFlHb for ABZ and its metabolites were determined from spectral titration of the saturation curve of the ferric heme using a Shimadzu UV-1800 UV/Visible Scanning Spectrophotometer. Ligand binding was determined by the difference in absorbance (Δ*A*) at 416 nm between the enzyme-ligand complex and the free enzyme. These experiments were performed at 37°C in 50 mM potassium phosphate buffer (pH 7.5) using 0.85 μM gFlHb and 0 to 5 μM of ABZ, with the ligand-free solution of gFlHb recorded as the baseline. A control experiment done with DMF, in which the ABZ stock solution was prepared, did not present any peaks in the spectrum that would interfere with the measurement. For comparison, a difference spectrum titration of gFlHb was also performed with imidazole.

NADH oxidase activity assays of gFlHb were carried out in a Cary 60 UV-VIS spectrophotometer. Reactions were monitored by following the NADH oxidation (the decrease in absorbance at 340 nm over time) at 37°C under aerobic conditions. Enzyme assays contained 150 μM NADH and 0.37 μM gFlHb in air-equilibrated 50 mM sodium phosphate buffer at pH 7.5 in a total volume of 1 mL, and the reactions were started by adding 1 μM FAD. The NADH oxidase activity of gFlHb in the presence ABZ (0–5 μM), ABZSO and ABZSOO (0–40 nM) was measured in a similar manner. In these experiments an additional control was included that contained the highest concentration of the DMF carrier but no drug. Activity was determined under initial velocity conditions during which the accumulation of products was minimal, and the activity was linear respect to the protein concentration. NADH oxidation rates were calculated from the absorbance changes over time by using an extinction coefficient of 6.22 mM^-1^ cm^-1^ for NADH. The NADH oxidase activity assay of gFlHb was also performed in the presence of 5U superoxide dismutase (SOD) and 13 U catalase (CAT) in order to scavenge the production of superoxide [50]⁠. To determine the efficacy of SOD in these experiments, enzyme assays similar to those described above were performed in the presence of 25 mM nitroblue tetrazolium (NBT) and 1 or 5 U of SOD, and the change in absorbance was determined at 550 nm in a Varioskan microplate reader (ThermoFisher). It was determined that with 5U SOD present, no superoxide was detected (S6 Fig) and this amount was used for further experiments.

UV-visible spectroscopy was also used to identify the production of ROS (particularly superoxide) and ABZSO, the latter by detection of its characteristic peak at 288–293 nm in the presence of gFlHb or the superoxide-generating system of xanthine/ xanthine oxidase (XO) as reported by Beauchamp and Fridovich [51]⁠. These assays were carried out using a SmartSpec 3000 spectrophotometer (BioRad) using quartz cuvettes of 1 cm path. Superoxide (O_2_^-^) production from gFlHb and XO was determined with NBT in reaction mixtures containing 0.176 μM enzyme in 50 mM sodium phosphate buffer equilibrated with air at pH 7.5, 150 μM NADH, 1 μM FAD (for gFlHb), or 150 μM xanthine (for XO) in a total volume of 1 mL. These assays were carried out in the presence or absence of 150 μM ABZ. Measurements of the absorption spectrum in the reaction mixture with 1.0 μM enzyme were made by scanning at the range 200–400 nm. At the end of the reactions (30 minutes), ABZSO at 150 μM (final concentration) was added and samples were read again to verify the identity of this compound as a reaction product.

### Detection and quantitation of ABZ and its metabolites

Enzyme assays containing gFlHb (6.6 μM) and ABZ, or enzyme-free controls were run for about 20 minutes at pH 7.5, 150 μM NADH, 1 μM FAD, and 37°C. The contents were collected in 1.5 mL microtubes and stored at -70°C until analysis. Extraction of ABZ and its metabolites was carried out as follows: 300 μL of gF1Hb reaction sample were transferred to an assay tube and spiked with 100 μL of carbamazepine solution (200 ng/mL) as an internal standard (IS). Then, 5 mL of ether-dichloromethane-chloroform (60:30:10, *v/v*) were added and samples were vortex-mixed for 5 min. After centrifugation at 750 x *g* for 20 min, samples were frozen at -70°C for 20 min and the upper organic phase was transferred to clean glass tubes and evaporated to dryness at 45°C with a stream of nitrogen. Finally, samples were reconstituted with 100 μL of the mobile phase (methanol:formic acid 70:30, *v/v*), and injected into the chromatographic system. The assay range was 2.5–500 ng/mL for ABZ, 15–500 ng/mL for ABZSO and 7.5–200 ng/mL for ABZSOO.

Levels of ABZ, ABZSO and ABZSOO were determined by liquid chromatography-mass spectrometry (LC-MS/MS) using the modified method of González-Hernández et al., 2012 [52]⁠. The LC-MS system consisted of an Agilent 1100 system equipped with a quaternary pump and an autosampler (Palo Alto, CA, USA). Mass spectrometric detection was performed on an ABSciex 3200 QTrap mass spectrometer (ABSciex, Darmstadt, Germany). Chromatographic separation was performed using a Gemini C18 analytical column (150 x 4.6, 5 μm, Phenomenex C18 ODS) attached to a pre-column (Phenomenex C18 ODS). The mobile phase was composed of methanol:formic acid (70:30, v/v) at a 0.7 mL/min flow rate. Compounds eluting from the column were analyzed in the positive ionization mode, with electrospray ionization (ESI) using the following mass spectrometer settings: curtain gas 20 psi, ion spray voltage 5500 V, capillary temperature 600°C, nebulization gas 50 psi, heating gas 40 psi. Quantification was performed using multiple reaction monitoring (MRM). Table 1 shows the optimum MS parameters for ABZ, ABZSO, ABZSOO and CBZ.

**Table 1:** Optimized MS/MS parameters for ABZ, ABZSO, ABZSOO and CBZ.

Analyte	MRM transition*(m/z)*	DP^1^ (V)	EP^2^ (V)	CE^3^ (V)	CXP^4^ (V)
**ABZ**	266.3 → 234.0	34	4.5	25	3.0
**ABZSO**	282.0 → 240.0	20	5.0	16.0	3.0
**ABZSOO**	298.2 → 266.2	27	4.7	25.4	3.0
**CBZ**	237.2 → 194.1	43	4.0	22	3.0

^1^DP: Declustering potential

^2^EP: entrance potential

^3^CE: collision energy

^4^CXP: collision exit potential.

Data acquisition and processing were performed using the Analyst software version 1.6.2 (Applied Biosystems, Foster City, CA, USA) and quantitative analysis was performed using MultiQuant software version 3.0 (Applied Biosystems, Foster City, CA, USA).

### Western blotting

Total protein extracts of 1.5 x 10^7^ trophozoites from non-transfected, empty vector or FLAG-tagged-gFlHb transfected cultures were suspended in 500 μL of PBS, 1.5% Triton X-100, and were lysed by sonication. Protein concentration was determined using a Pierce BCA Protein Assay Kit (Thermo Scientific) and the absorbance was measured at 562 nm in a microplate reader (BioRad). Then, 50 μg of protein samples were separated by SDS-PAGE in 10% polyacrylamide gels under denaturing conditions. Western blots were performed by electrophoretic transfer of SDS-PAGE-separated proteins onto nitrocellulose membranes for 1.5 h at 300 mA in the cold. Membranes were blocked for 1 h at room temperature with a solution 5% nonfat dried milk and 0.1% Tween 20 in TBS. The membranes were then incubated overnight at 4°C with mouse monoclonal antibody α-FLAG M2 (Sigma-Aldrich Co., St. Louis, MO, USA) at a 1:100 dilution and anti-actin at 1:1000 dilution, prepared in blocking solution. The membranes were then washed three times with 0.1% Tween 20 in TBS and incubated with horseradish peroxidase-conjugated goat anti-rabbit IgG secondary antibody (Promega) diluted 1:30000 in blocking solution, for 1 h at 37°C and constant shaking. Chemiluminescence detection was performed with an Amersham Western Blotting ECL detection kit (GE Healthcare) according to manufacturer´s instructions.

### Analysis of gFlHb expression by RT-PCR

RNA was extracted from 1 x 10^7^ trophozoites of WB strain following the TRIzol protocol (Life Technologies). The concentration and purity of RNA was quantified using a NanoDrop ND-2000 device (Thermo-Scientific, Wilmington, USA), and its integrity was verified in a 1.5% (*w/v*) agarose gel under denaturing conditions. The RNA samples were treated with DNaseI (Amplification Grade, Life Technologies) for 30 min at 37°C. RNA (8 μg) was used in cDNA synthesis with oligo (dT) oligonucleotides and SuperScript II Reverse Transcriptase (Invitrogen). cDNA (1 μg) was used in PCR reactions with the following gFlHb primer set: Forward primer (EcoRV site in lower case), 5’ gatatcATGACGCTTTCCGAAGA 3’; Reverse primer (NotI site in lower case), 5’ gcggccgcTAATGGGAGG 3’. For the PCR reaction, an initial denaturation at 95°C for 5 min was followed by 30 cycles of denaturation at 94°C for 30 s, annealing at 60°C for 30 s, and elongation at 72°C for 1 min; these cycles were followed by a final extension step for 7 min at 72°C. Amplification of ubiquitin transcript (Forward primer 5’ GATCTTCGTCAAGACTCTCACCG 3’; Reverse primer 5’ TAGTTACCACCACGGAGGCG 3’) was used as housekeeping gene control. The amplified fragments were analyzed on 1.2% agarose gels stained with ethidium bromide, which were analyzed using ImageJ 1.8.0 software to quantify the optical density of the PCR bands from each sample. The results were expressed as relative densities in comparison to the optical density of the background and were normalized to the expression of the housekeeping gene for ubiquitin.

### Transfection of ABZ-susceptible and ABZ–resistant trophozoites

The gene sequence of gFlHb (GL50803_15009) was obtained from the *Giardia* DataBase corresponding to strain WB clone C6 (https://giardiadb.org). The coding sequence was PCR-amplified from *Giardia* genomic DNA using the following primer set: Forward primer (EcoRV site underlined) 5´ GATATCATGACGCTTTCCGAAGA 3´; Reverse primer (NotI site underlined) 5´GCGGCCGCTAATGGGAGG 3´. PCR assays were performed using Pfu polymerase, and fragments were cloned into the pJET1.2/blunt vector (Thermo Scientific) according to the manufacturer’s instructions. The amplicon was digested with EcoRV and NotI at 37°C and ligated to the *Giardia* expression vector pAC (kindly donated by Dr. Staffan Svärd, Uppsala University, Sweden). The vector construct was then used to transform *E*. *coli* BL21 (DE3). Colonies that incorporated the plasmid were screened by colony PCR and restriction enzyme digestion. Plasmids were sequenced using a BigDye Terminator v3.1 Cycle Sequencing Kit (Applied Biosystems) according to manufacturer´s instructions. After sequence verification, transfection of ABZ-susceptible (WB and WB-DMF) and ABZ-resistant (R1.35, R8.0 and R250) trophozoites was performed by electroporation on a Gene Pulser XCell electroporator (Bio Rad) using 50 μg of plasmid or the empty vector and 1 x 10^7^ trophozoites according to the protocol of Singer et al. (1998) [53]⁠. Twenty-four hours after transfection, puromycin hydrochloride (Sigma-Aldrich) was added to cultures at 25 μg/mL concentration. Cultures with confluent trophozoites (non-transfected, pAC or pAC-gFlHb transfected) were harvested by incubation on ice for 30 min. Suspended motile trophozoites were counted and identical number of trophozoites (1 X 10^6^) were inoculated in 4.5 mL screw capped vials in the culture media described above, and increasing concentrations of ABZ, MTZ, S-Nitrosoglutathione (GSNO) or a solvent control (DMF) were added. The vials were then incubated under anaerobic conditions at 37°C for 24 h and trophozoites viability was determined by Trypan blue exclusion.

### Statistical analyses

For statistical analysis of the results obtained on drugs treatment assays, on the determination of gFlHb expression levels, and of the IC_50_ values for ABZ, the Student´s *t*-test and ANOVA in the GraphPad Prism 6 program (GraphPad Software, San Diego, Ca. USA) were used.

## Results

### ABZ-resistant lines are also resistant to ABZ metabolites

**Fig 2:**
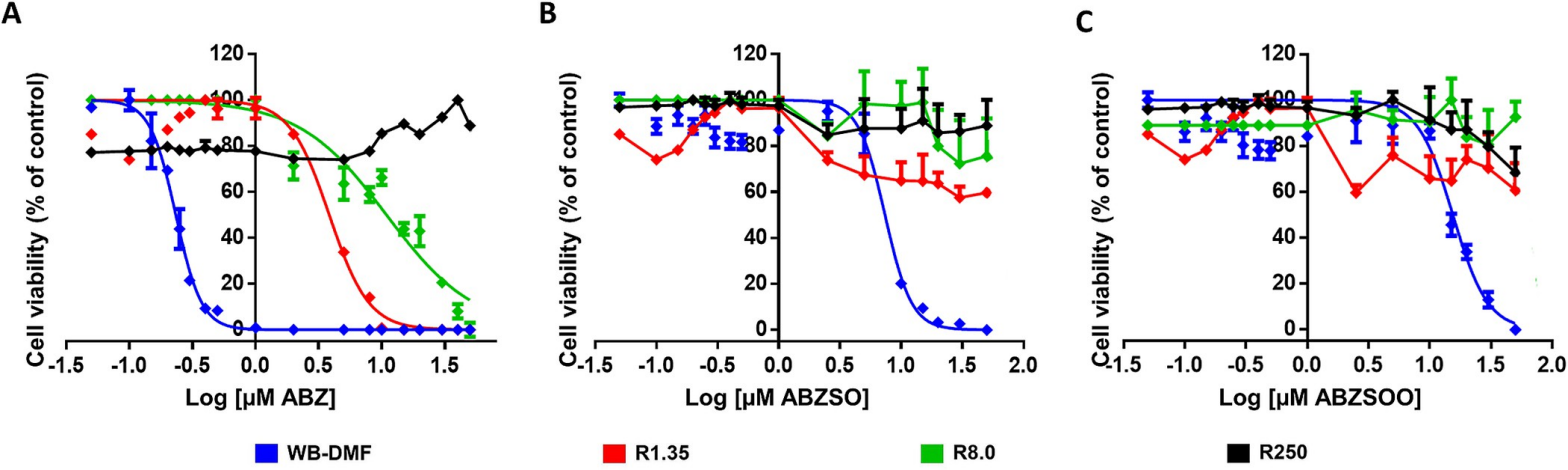
Concentration-viability curves of ABZ-sensitive and ABZ-resistant *G*. *duodenalis* trophozoites to ABZ and its metabolites. Cell viability of trophozoites exposed to increasing ABZ (A), ABZSO (B) and ABZSOO (C) concentrations (shown in logarithmic scale) for 24 h was determined by Trypan blue exclusion. The IC_50_ values correspond to n = 3 independent experiments, respectively. Standard deviations are shown for each drug concentration and for each IC_50_ determined.

Since ABZ is a drug with an intrinsic ability to bind β-tubulin and thereby affect microtubule functions in *Giardia* [6,7]⁠, it was initially assessed whether its ABZSO and ABZSOO metabolites also possessed giardicidal activities. We evaluated the effect of the exposure of ABZ metabolites on ABZ-sensitive and ABZ-resistant trophozoites. WB-DMF trophozoites exposed to ABZ showed half maximal inhibitory concentration values (IC_50_) as expected (0.21 ± 0.02 μM), but there were increases of 36.6-fold in IC_50_ with ABZSO (7.70 ± 0.60 μM) and 79-fold with ABZSOO (17± 2 μM) (Fig 2) in these cultures. In contrast, ABZ-resistant trophozoites (R1.35, R8 and R250) displayed, much higher IC_50_ values as compared with the WB-DMF clone upon exposure to ABZ (3.8 ± 0.62 13 ± 2 and 580 ± 90 μM respectively). Interestingly, exposure of ABZ-resistant trophozoites to ABZSO and ABZSOO did not cause change in viability with the concentrations used in the ABZ-susceptible trophozoites (Fig 2B and 2C). These data indicate that ABZ metabolites have a diminished giardicidal activity compared to ABZ on ABZ-sensitive parasites; moreover, ABZ-resistant trophozoites were unaffected by ABZ metabolites as did ABZ-susceptible ones.

### Hemin modulates the expression of gFlHb

**Fig 3:**
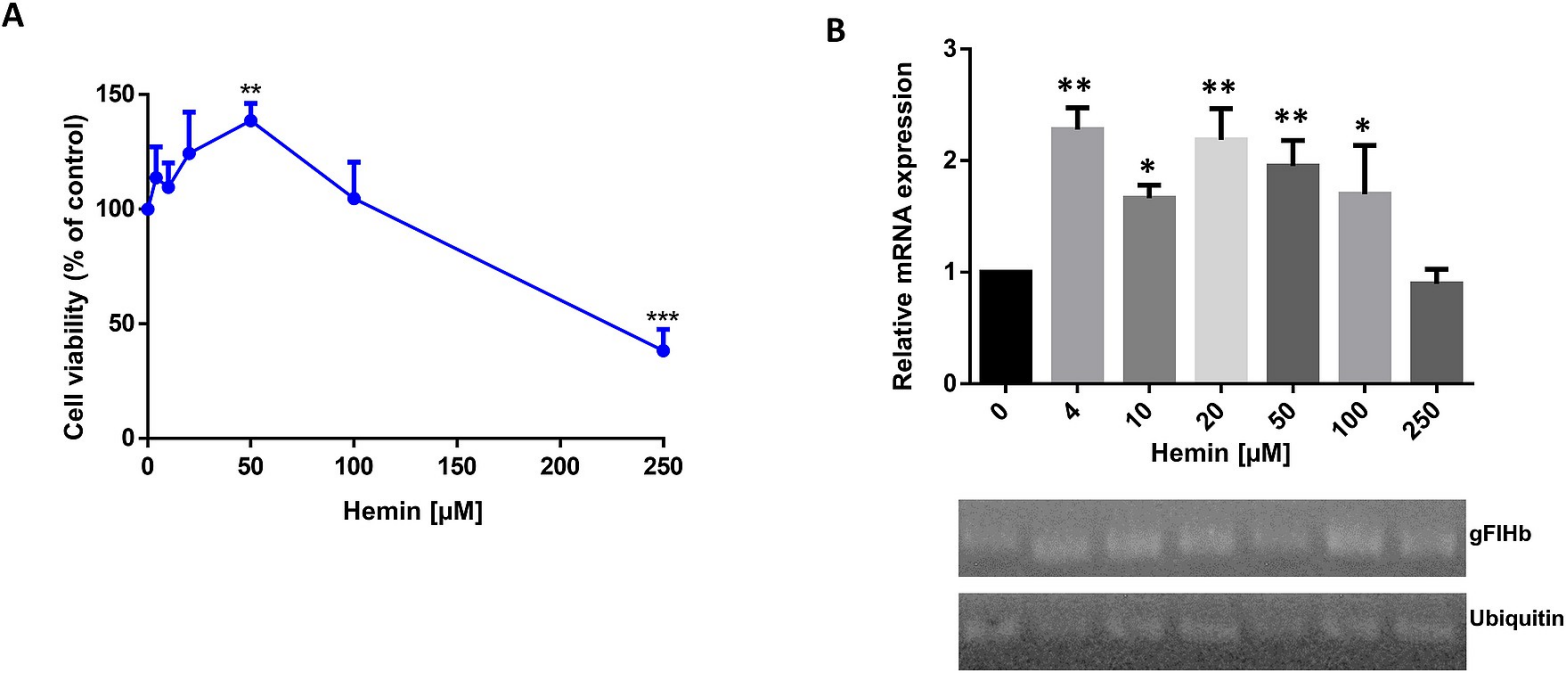
Effect of hemin on cell viability and gFlHb expression in *G*. *duodenalis* WB-DMF trophozoites. (A) Cell viability of trophozoites exposed to increasing hemin concentrations. (B) RT-PCR analysis of gFlHb transcript levels. The values represent the ratio of the optical densities of the PCR bands of gFlHb and internal standard ubiquitin. Densitometry values are the mean corresponding to n = 3 independent experiments. An asterisk denotes a statistically significant difference (* p < 0.05, ** p < 0.01 and ***p < 0.001), as determined by ANOVA test.

To assess whether hemoproteins could be involved in ABZ resistance in this parasite, we evaluated the cell viability of trophozoites exposed to hemin, a known cofactor required for hemoprotein activity. First, the response of WB-DMF trophozoites to increasing concentrations of hemin was evaluated; our results showed a significant increase in survival (138%) at 50 μM hemin compared to the control cultures with no hemin added. Trophozoite survival decreased at concentrations greater than 100 μM (Fig 3A). From these data and considering that hemin was not freely soluble at concentrations exceeding 100 μM, it was assumed that soluble hemin is not toxic for trophozoites, thus, an IC_50_ was not calculated.

As noted above, *Giardia* has at least five hemoproteins, four of which are cytochrome *b*_5_ isoforms that act as electron shuttles, and one flavohemoglobin (gFlHb) with nitric oxide dioxygenase activity [14,33]⁠. It was found that gFlHb transcript levels increased about twofold in the presence of 4 to 100 μM hemin (Fig 3B), but decreased to basal level at 250 μM hemin, in agreement to the survival rates of trophozoites at different hemin concentrations. These results indicate that the expression of gFlHb is modulated by the concentration of hemin in the extracellular milieu. The absence of flavohemoglobin homologs in hosts including humans, together with its known dioxygenase activity, led us to hypothesize that gFlHb could be related to the oxidation of ABZ to ABZSO and ABZSOO in *G*. *duodenalis*.

### Molecular modeling of gFlHb

The results obtained in hemin susceptibility and gFlHb gene expression assays led us to propose gFlHb as a candidate for the oxygenase activity needed for ABZ conversion into its sulfoxide metabolite. It is the only heme-containing dioxygenase in *Giardia*, and its catalytic site can accommodate a variety of azole-based inhibitors as well as analogs of these as benzimidazoles (e.g. ABZ) [34,54–57].⁠

**Fig 4:**
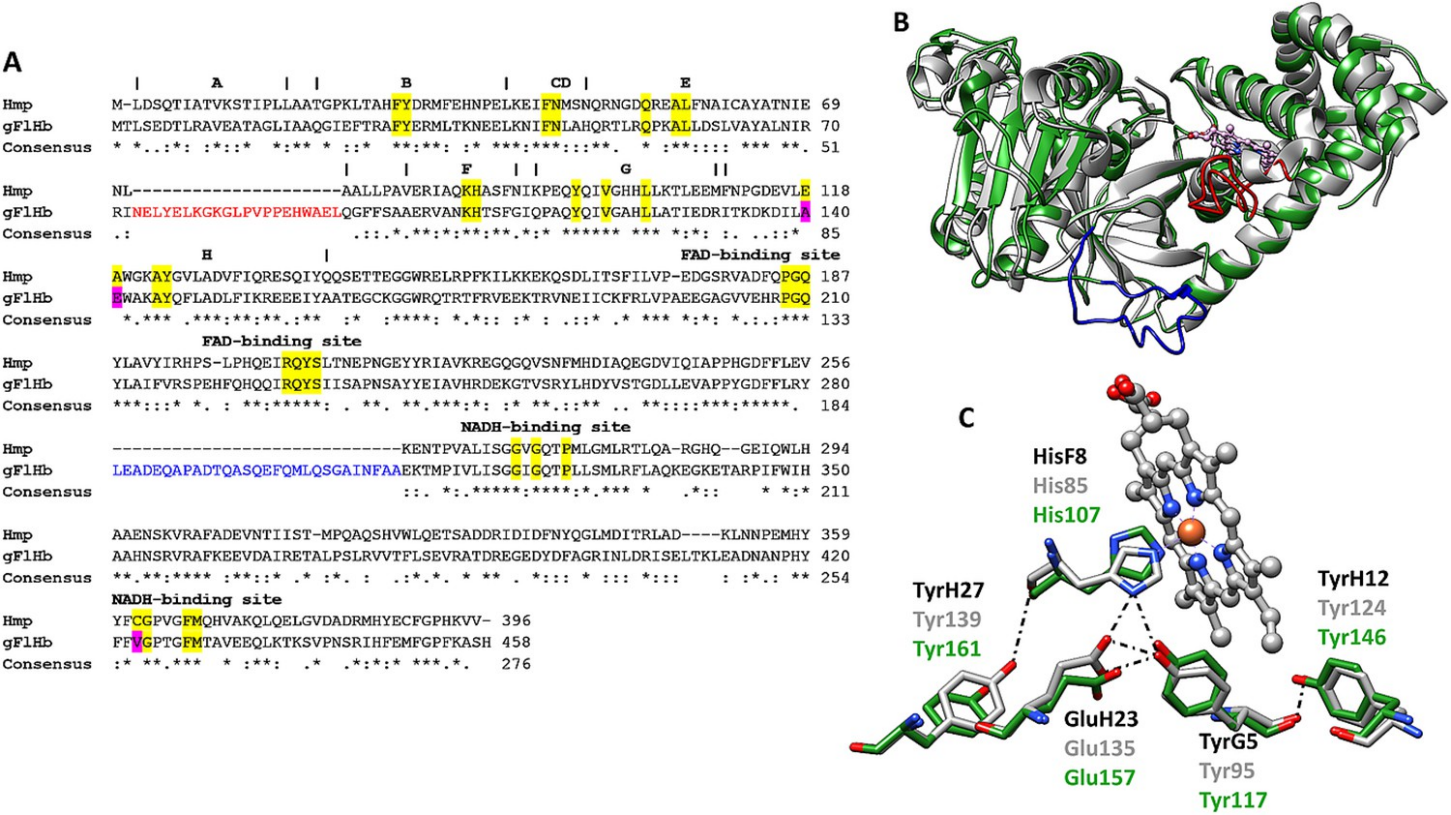
Sequence alignment and protein structure alignment of flavohemoglobins of *G*. *duodenalis* (gFlHb) and *E*. *coli* (Hmp). (A) Sequence alignment of gFlHb and Hmp. The conserved residues in the heme and the reductase domains are highlighted in yellow and differences present in gFlHb are highlighted in pink. The additional sequence inserts in the globin and FAD domains in gFlHb are highlighted in red and blue, respectively (Rafferty et al. 2010) [30]⁠. Asterisks indicate identical residues, while colons and periods indicate conserved residues. (B) Structural overlap of gFlHb (green) and Hmp (gray; ID PDB: 1GVH). (C) Proximal site of hydrogen bonding pattern of consensus FlHb (black), gFlHb (green) and Hmp (gray).

The sequence alignment of gFlHb with other flavohemoglobins identifies the distinct heme and FAD-binding domains at the N- and C-termini, respectively. The closest sequence similarity of gFlHb to a FlHb of known structure corresponds to Hmp of *E*. *coli* (55.2% sequence similarity and 38.7% sequence identity, Fig 4A). The reductase domain of gFlHb displays two conserved motifs: a RXYS motif of the FAD binding site and a GXGXXP motif of NADH binding site [58]⁠. This allowed the structural modeling of gFlHb using the I-TASSER server [59]⁠, which performs structural alignments between query sequences and known templates in the PDB library. The model validation was carried out with PROCHECK [60]⁠ and model quality was evaluated using QMEAN [61]⁠ and ProSA-Web [44]⁠ servers. In these evaluations, the scores obtained were of -11.09 and -8.08, respectively (S1 Fig). Although the QMEAN value is below the values of the X-ray structures, the ProSA-Web score qualified it as a good model. The Ramachandran plot for the gFlHb model indicates that 71.0% of the residues are located in most favored backbone geometries, 21.2% in allowed regions, 5.9% in generously allowed and only 2.0% in disallowed regions. Consistent with their sequence similarity, molecular modeling of gFlHb showed a high structural similarity to *E*. *coli* Hmp (PDB: 1GVH) with a TM-score of 0.836 and a root-mean-square-deviation (rmsd) of 1.44 Å (Fig 4B), indicating *bona-fide* similarity between these two structures. Unlike other flavohemoglobins, gFlHb contains two large sequence insertions of unknown function: a 21 residue insert in the N-terminal globin domain, and a 29 residue insert in the C-terminal FAD-binding domain [30]⁠. These inserts are predicted to lie adjacent to each other, and structural tunnel analyses suggest that they could be involved in allowing substrate entry (S2 Fig). gFlHb also exhibits a conserved hydrogen bonding network within the proximal site created by HisF8-TyrG5-GluH23, which has been proposed to provide an imidazolate character and confer strong electronic push for activation of heme-bound oxygen (Fig 4C) [62,63].⁠

### Molecular docking analyses

**Fig 5:**
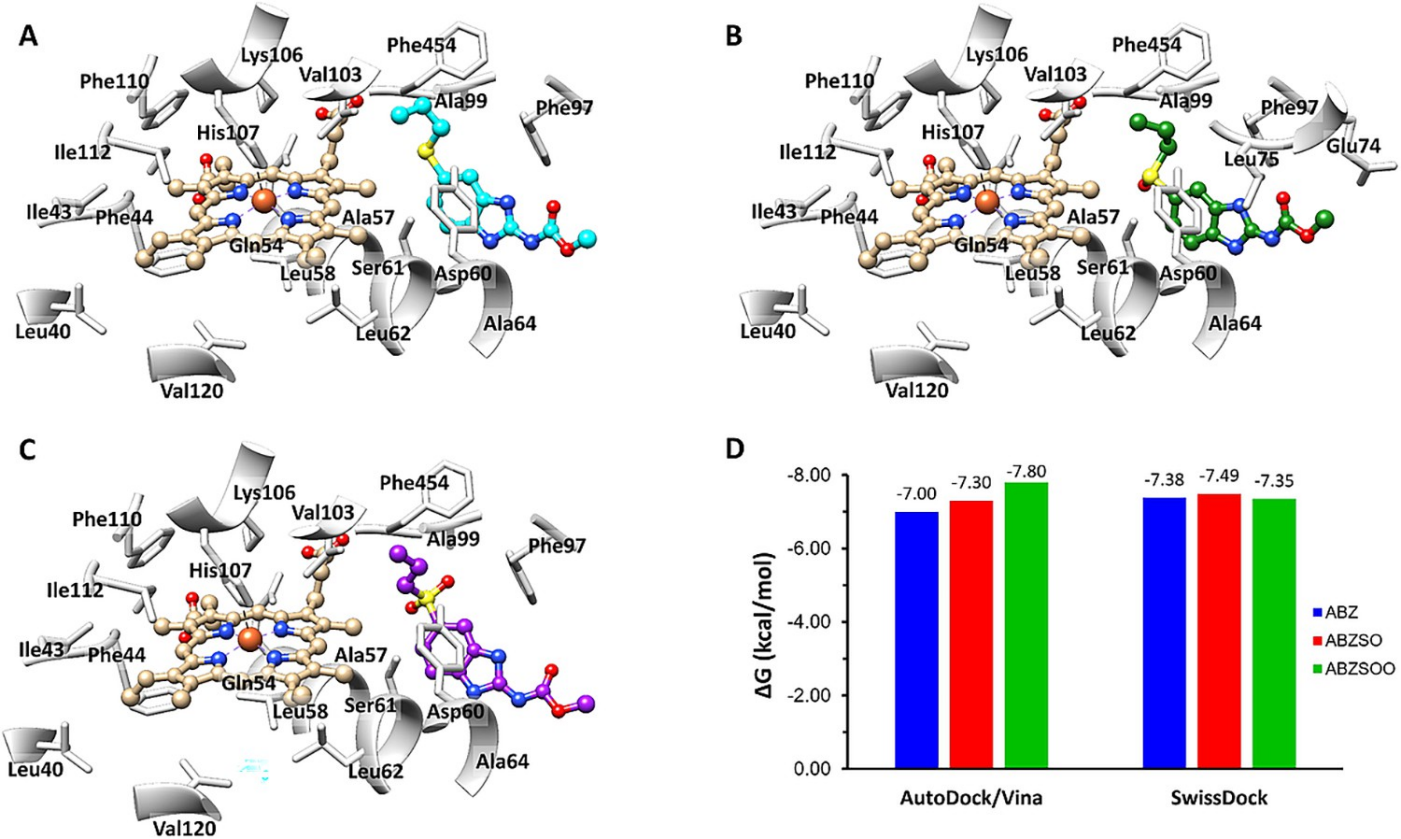
Docking positions and molecular interactions of drugs at the active site of gFlHb. Optimal dockings for (A) Albendazole (RMSD = 3.9 Å), (B) Albendazole sulfoxide (RMSD = 3.0 Å), (C) Albendazole sulfone (RMSD = 2.8.2 Å) were obtained and are shown with residues of the catalytic site. (D) Predicted affinities for ABZ, ABZSO and ABZSOO to gFlHb using AutoDock/Vina and SwissDock.

**Fig 6:**
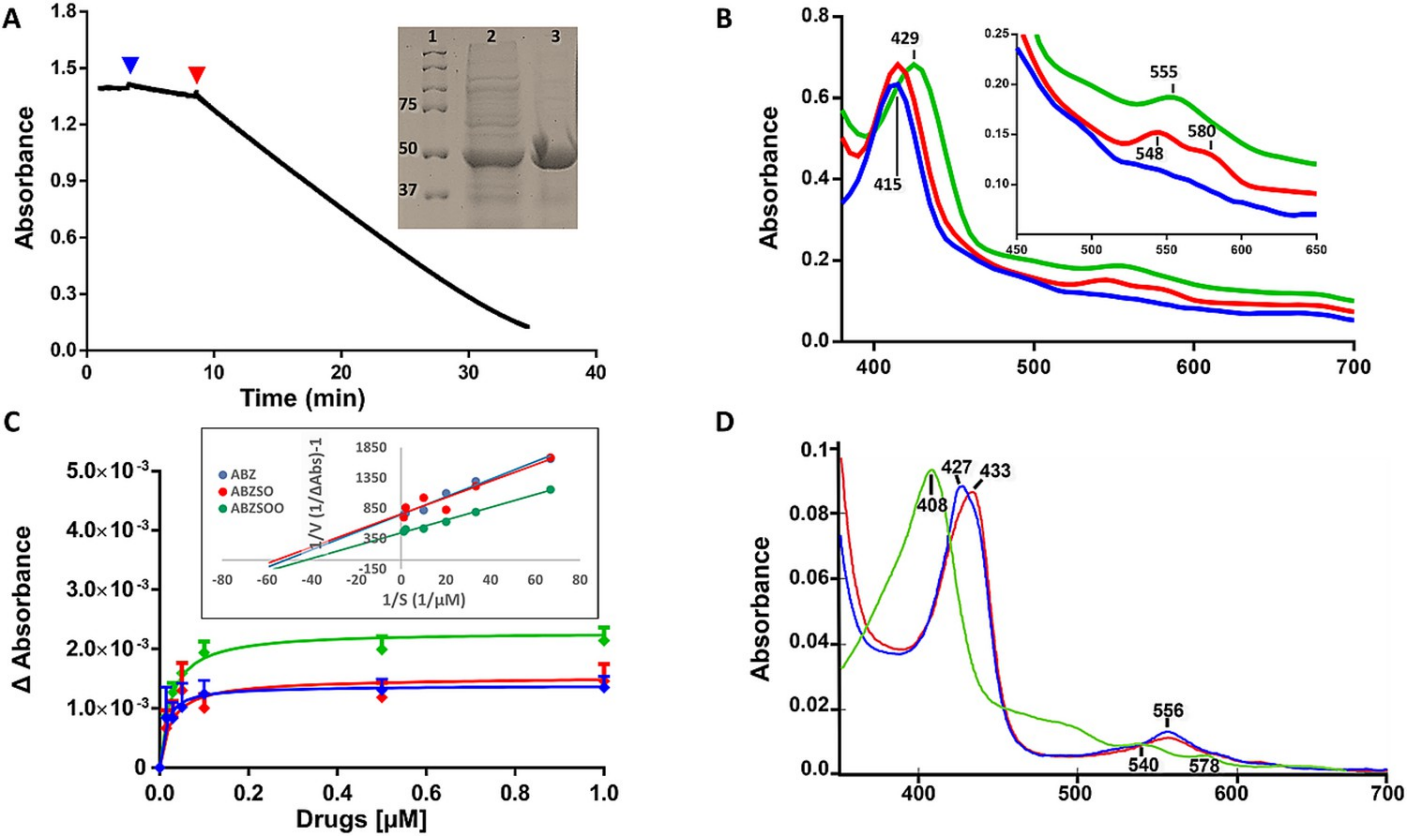
NADH oxidase activities of recombinant gFlHb. (A) The reaction was performed in 50 mM potassium phosphate buffer by adding 160 μM NADH at time zero, after 0.37 μM gFlHb (blue arrow) was added to reach a stable baseline with negligible spurious reaction. Afterwards, 1 μM FAD (red arrow) was added to initiate the full reaction. The NADH consumption was monitored by the decrease in absorbance at 340 nm. Inset shows the purified gFlHb protein detected by 10% SDS-PAGE and Coomassie blue staining: 1) Molecular weight markers; 2) Cell lysate of *E*. *coli* overexpressing gFlHb; 3) Purified gFlHb in 53 kDa. (B) Spectral characteristics of gFlHb. Spectral traces of 6.6 μM gFlHb in the ferric state (blue line), NADH-reduced (red line) and with ABZ (green line). (C) Saturation curves of ABZ and its metabolites under aerobiosis. Increasing concentrations of ABZ (blue), ABZSO (red) and ABZSOO (green) were added to oxidized gFlHb (0.85 μM), and coordination was followed by monitoring the absorbance difference (Δ*A*) between the drug- complexed and the free enzyme at 416 nm. The experiments were performed at 37°C in 50 mM potassium phosphate buffer (pH 7.5). Inset double-reciprocal plots showing a lineal behavior, used to calculate the *K*_*d*_ values. (D) Spectral characteristics of gFlHb under anaerobiosis. Spectral traces of 0.65 μM gFlHb in the ferric state (black), reduced with sodium dithionite (red) and with ABZ (blue). These experiments were performed at 37°C in 0.1 M Bis-Tris (pH 6.5) and measurements were obtained using a Shimadzu UV-1900i spectrophotometer.

To provide *in silico* support to the hypothesis that gFlHb may participate in ABZ biotransformation, molecular docking simulations were performed with ABZ, ABZSO, ABZSOO and azole inhibitors as ligands of gFlHb using Autodock Vina and SwissDock. These analyses showed that ABZ, ABZSO and ABZSOO docked at positions that are favorable for its interaction with the active site of gFlHb, (Fig 5). The mean standard error for Autodock Vina was 2.8 kcal / mol; however, ABZ, ABZSO and ABZSOO did not display significant difference between Autodock (-7.0, -7.3 and -7.8 kcal/mol) and SwissDock (-7.4, -7.5 and -7.4 kcal / mol) server calculations (Fig 5D) which would bind with the same affinity previously observed (Fig 6C, see below). On the other hand, ABZ, ABZSO and ABZSOO are present in a conformation where the sulfur group was oriented towards heme, thereby suggesting interaction with the oxygen atom. The azole inhibitors econazole, ketoconazole and miconazole, had good efficient energy values and had little energy variations between Autodock Vina (-9.6, -9.5 and -8.5 kcal / mol) and SwissDock (-8.27, -8.48 and -8.24 kcal / mol) servers´s (S4 Fig). Thus, all the azole inhibitors are present in a conformation in which the imidazole ring is oriented towards the heme group. These data suggest that azole inhibitors could target gFlHb, thereby inhibiting its activity as it has been observed with other flavohemoglobins [34,57]⁠. Based on these molecular predictions using the predicted protein structure, the next approach was to search for the ability of gFlHb to bind and metabolize ABZ.

### Protein expression and spectral characteristics of recombinant gFlHb

Recombinant gFlHb was purified by nickel affinity chromatography. SDS-PAGE analysis confirmed the presence of the protein at a 53-kDa band (Fig 6A, inset). This result indicates that the production of recombinant gFlHb protein is optimal in 24 h cultures. The reddish-brown appearance and NADH oxidase activity of the protein (Fig 6A) indicated that the enzyme is able to fold and bind heme efficiently. Based on the pyridine-hemochrome assay, each mole of protein presented 0.65 to 0.89 equivalents of heme.

UV-visible absorption spectroscopy was carried out to determine the spectral properties of recombinant gFlHb. The spectra of the purified protein indicated that it predominantly exists in the oxidized (ferric) state under an aerobic environment and appears to be a mixture of high- and low-spin species based on the Soret peak at 415 nm while the Q bands are absent (Fig 6B). This Soret peak at 415 nm could indicate the presence of imidazole remaining from the purification process of the recombinant gFlHb. When gFlHb was reduced by NADH, a pair of Q bands appears at 548 nm and 580 nm, indicating that the protein was predominantly in the oxygen-bound state (Fig 6B). The spectrum of the NADH-reduced gFlHb with ABZ caused the Soret peak to shift to 425 nm, with a new band appearing at 555 nm; this species seems to be a mixture of high- and low-spin species based on the asymmetrical Soret peak.

Such spectral shifts were also observed in the presence of ABZSO and ABZSOO, which indicates possible heme-iron coordination with the imidazole group of these drugs. Imidazole was used as a positive control since it is known to interact with the heme group changing the spectral profile, while acetylsalicylic acid was used as a negative control (Table 2).

**Table 2:** Spectroscopic properties of NADH-reduced gFlHb under aerobic conditions in the presence of albendazole and other ligands.

Axial ligand	Absorption maximum (± 0.3 nm)	λ_vis_
Fe(II)-O_2_	415	548/580
Fe(II)-ABZ	429	555
Fe(II)-ABZSO	430	555
Fe(II)-ABZSOO	430	555
Fe(II)-Imidazole	430	555
Fe(II)-Aspirin	415	-

The saturation curves of ferric gFlHb with ABZ and its metabolites as well as the dissociation constant (*K*_*d*_) values for the drugs were obtained from the spectral titration curves at 416 nm. As shown in Fig 6C, data fitted well to a rectangular hyperbola, as further validated by Lineweaver-Burk linearization (Fig 6C inset), from which *K*_*d*_ values for ABZ (23 ± 9 nM), ABZSO (19 ± 8 nM) and ABZSOO (24 ± 4 nM) were calculated. Under anaerobic conditions, the recombinant enzyme could be reduced by dithionite to ferrous deoxy-gFlHb; a shift in the position of the Soret peak from 433.5 to 427 nm was observed in the absence and presence of ABZ respectively (Fig 6D). In this context, the interaction and high affinity of ABZ and its metabolites with gFlHb in both its ferric (S3 Fig) and ferrous states reflects a direct interaction with the active site and the possibility that these compounds are, besides NO, substrates of gFlHb.

### Albendazole increases NADH oxidase activity of gFlHb

**Fig 7:**
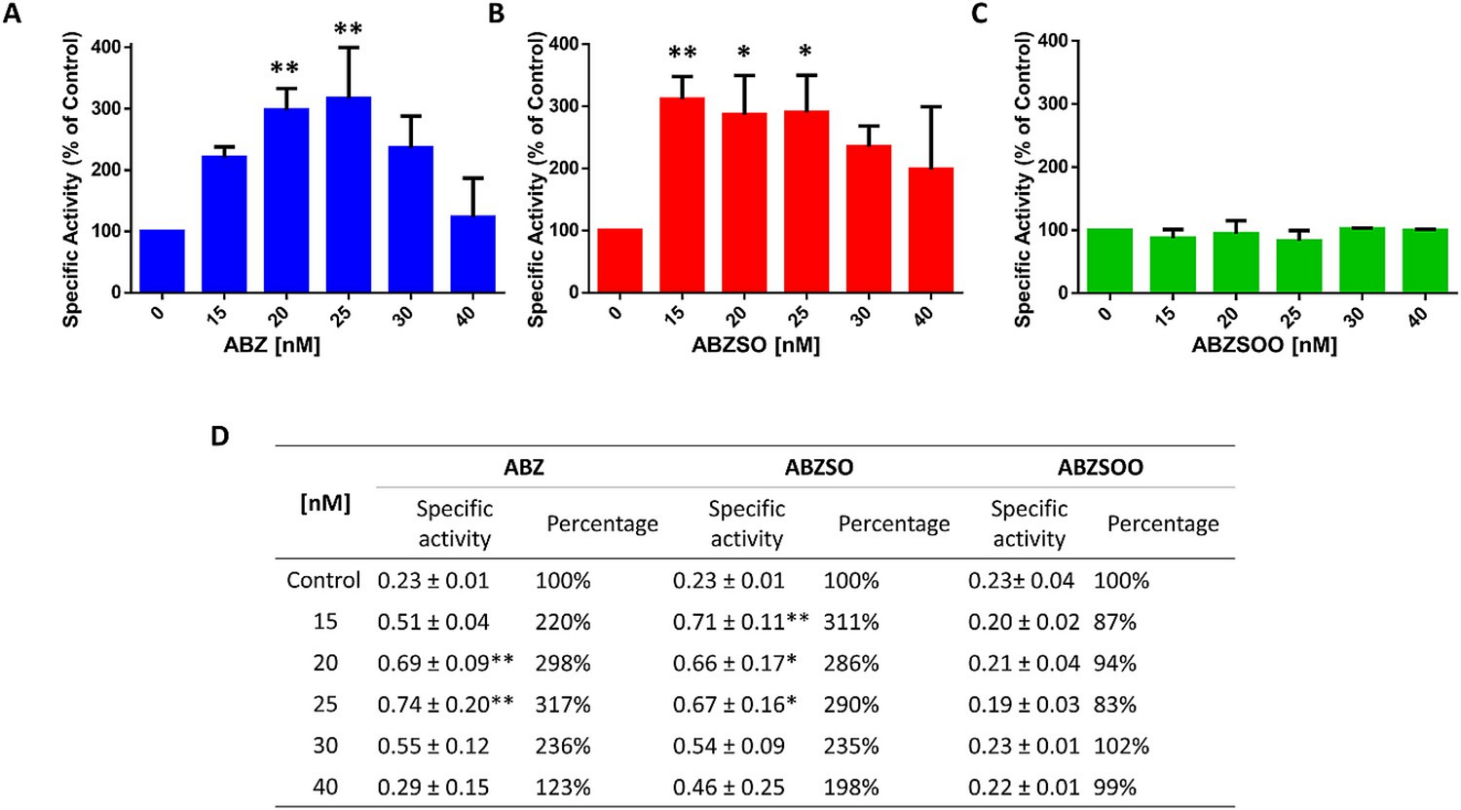
Relative specific activity of gFlHb in the presence of ABZ (A) and its metabolites sulfoxide (B) and sulfone (C). Enzyme activity was determined at 37°C in 50 mM potassium phosphate pH 7.5, 160 μM NADH, 0.65 μM gFlHb and 1 μM FAD was added to initiate the reaction. Values and errors (SE) shown are representative of n = 3 independent experiments (D). Absolute values of specific activities of gFlHb with ABZ, ABZSO and ABZSOO and percentage activity respect to control. The specific activity units are in μmol/min*mg. Asterisks denote statistical significant differences (* p < 0.05 and ** p < 0.01), as determined by ANOVA test.

*G*. *duodenalis* flavohemoglobin has basal NADH oxidase activity in the presence of molecular oxygen that is stimulated by the presence of nitric oxide [30]. W⁠e found that the presence of ABZ and ABZSO increased the NADH oxidase activity of gFlHb (Fig 7A and 7B). At 15–25 nM of ABZ or ABZSO, the NADH oxidase activity of gFlHb increased more than three-fold. A note of caution is in order on the gF1Hb activity. The net NADH consumed (approximately 20–30 μM, which derives from the absorbance change sustained at a fairly constant rate for the initial 20–25 min of reaction in the presence of 10–20 nM ABZ compared to in its absence, (S5A Fig) is more than 1000-fold greater than the ABZ added. From the data included in S5A Fig, a turnover number of 14.5 nmol product•nmol enzyme^-1^•min^-1^ was estimated with 0.37 μM gF1Hb for the initial 5 to 10 minutes of the reaction. If ABZ is a true substrate for gF1Hb, multiple oxidations should occur, which is unlikely unless other functional groups on ABZ, in addition to its thioether, are expected to be profusely oxidized in ABZ. Alternatively, ABZ and ABZSO might be gF1Hb substrates together with other potential unknown substrates present in the reaction assay, or ABZ and ABZSO are only stimulators of gFlHb activity. Furthermore, at concentrations greater than 25 nM ABZ, the NADH oxidase activity was no longer enhanced; although rate saturation is a common kinetic observation when using exceedingly saturating concentrations of substrate this is not the case here, as the enzyme concentration greatly exceeds the ABZ concentration. These data suggest a possible enhancing role of ABZ on the gFlHb NADH oxidase activity that could correspond to the proportion of gFlHb interacting with and overstimulated by ABZ. On the other hand, ABZSOO did not increase the NADH oxidase activity of gFlHb (Fig 7C), which would be expected since the sulfone is the product after complete oxidation of ABZ. The changes observed in the specific activity of recombinant gFlHb suggest that ABZ and ABZSO bind to gFlHb and may be partially transformed or have another role on the enzyme activity.

gFlHb is an enzyme able to produce reactive oxygen species (ROS) during its reaction cycle, particularly O_2_^-^ that could play a role in ABZ oxidation. To explore this possibility, the O_2_^-^ scavenging enzyme superoxide dismutase (SOD) was included in these assays. The inclusion of SOD and catalase in the assay did not alter the NADH oxidase activity of gFlHb (S5B–S5D Fig). In addition, the use of the ROS tracer NBT in the absence and presence of SOD confirmed that superoxide was the active free radical species produced from gFlHb activity (S6 Fig). In our subsequent experiments the possible role of superoxide in the oxidation of ABZ to ABZSO was assessed.

### Quantitation of ABZ metabolites produced by gFlHb

**Fig 8:**
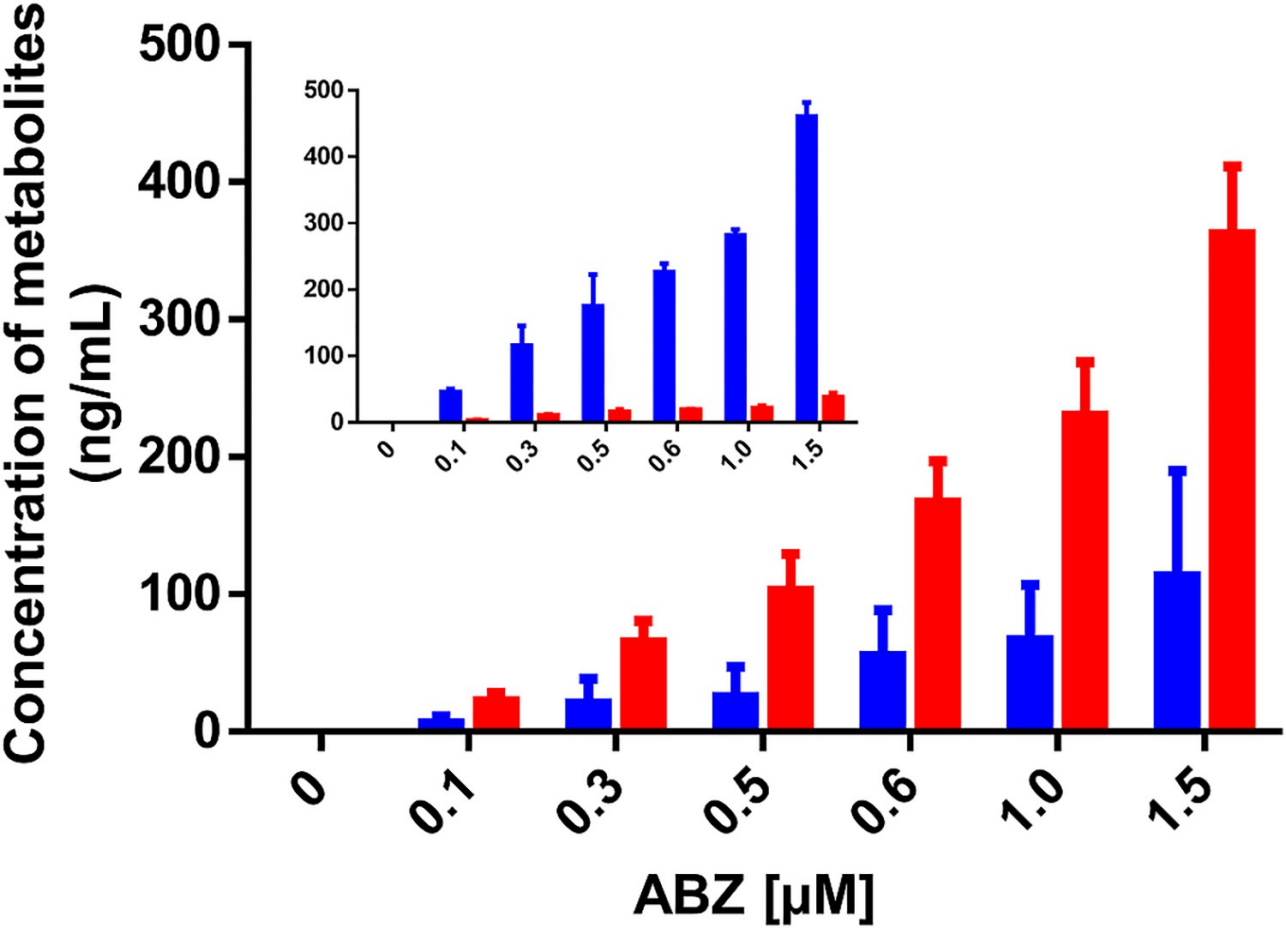
*in vitro* production of ABZ metabolites by recombinant gFlHb. The NADH oxidase activity assay of gFlHb (6.6 μM) was performed as described in Fig 7 in the presence of increasing ABZ concentrations. Remaining ABZ and the products of the reaction were quantified by HPLC-MS/MS. ABZ (blue) and its metabolite ABZSO (red). Values and errors (SE) shown are representative of n = 5 independent experiments. Inset shows ABZ and its metabolite concentrations in control reactions lacking gFlHb.

As ABZ increased the NADH oxidase activity of gFlHb, changed the UV-visible spectrum of this heme protein and is predicted to favorably dock with gFlHb, it seems likely that gFlHb metabolizes ABZ. The production of ABZ metabolites from gFlHb NADH oxidase activity was evaluated by LC-MS/MS. Under the chromatographic conditions used, retention times for ABZ, ABZSO and ABZSOO were 2.80, 3.06 and 3.14 min, respectively. With these experimental conditions it was possible to obtain a good resolution that allowed us to detect and quantify ABZ and its metabolites. The metabolite more reproducibly produced from ABZ was ABZSO (Fig 8). Although ABZSOO was detected in some samples, it did not exceed the lower limit of quantification which was set at 7.5 ng/mL and when detected, it was subtracted. ABZSO was detected from initial concentrations of ABZ, with a conversion rate (ABZSO/ABZ) of about 77% through all ABZ concentrations tested. For instance, at the highest ABZ concentration assayed (1.5 μM, Fig 8), approximately 1.39 μM ABZSO was detected (92% of the ABZ initially present) after a 20 minutes incubation reaction with 6.6 μM gF1Hb. From these values a gF1Hb turnover number of 0.01 nmol product•nmol enzyme^-1^•min^-1^ was estimated. This is much lower that the NADH oxidase activity data, in which a turnover number of 14.5 product•nmol enzyme^-1^•min^-1^ was estimated (S5A Fig). These two distinct enzyme activities suggest that gF1Hb promotes the conversion of ABZ to ABZSO as demonstrated by LC-MS/MS. Furthermore, ABZ oxidation to ABZSO was not observed when the enzyme was absent in the reaction medium (Fig 8 inset). Taken together, these data indicate that gFlHb is able to metabolize ABZ into ABZSO, although it does so at low rates.

### ABZ is oxygenated by gFlHb through oxidation by superoxide derived from its NADH oxidase activity

**Fig 9:**
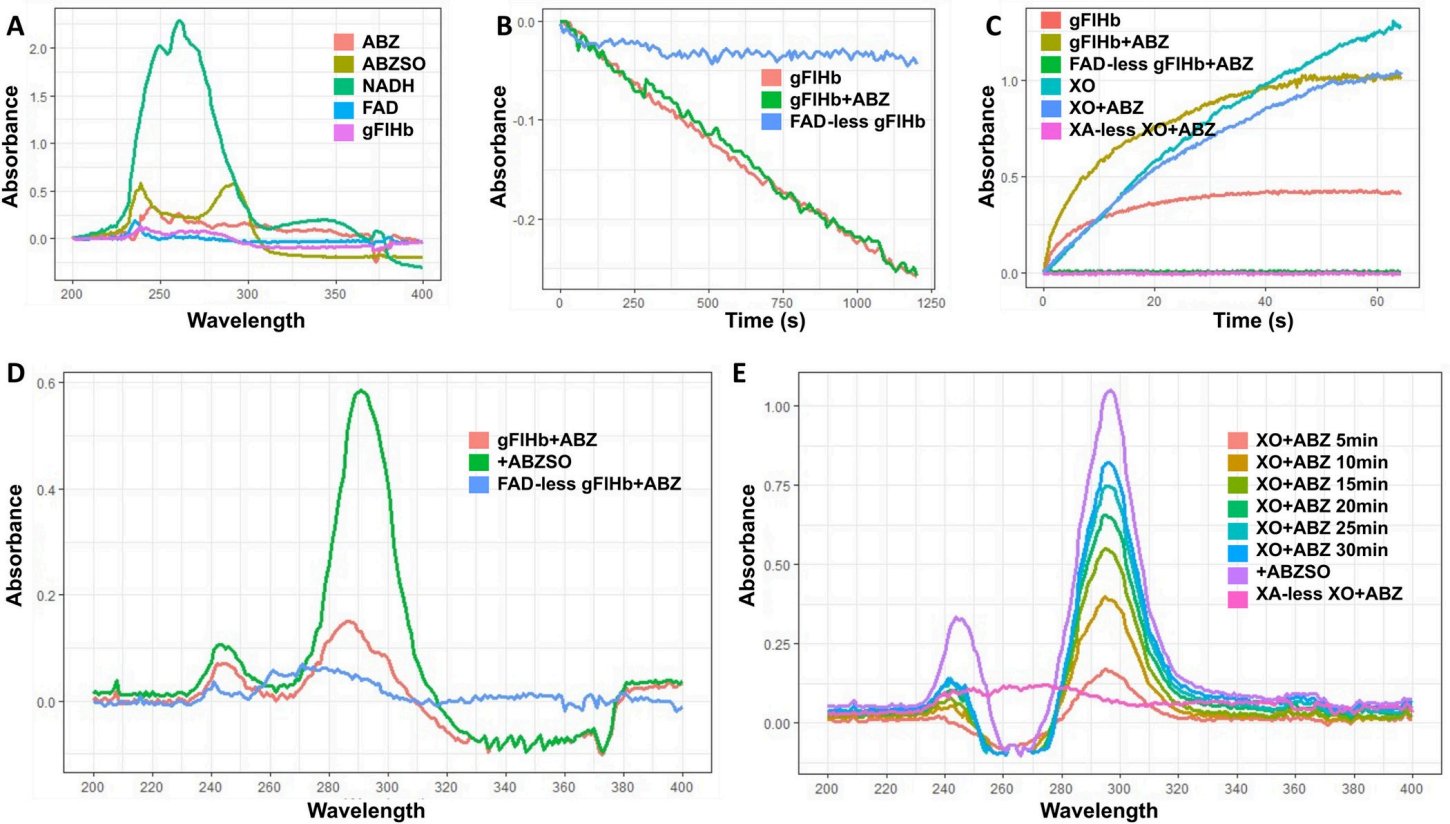
Spectral identification of ABZSO production by gFlHb. (A) Spectral characteristics of reagents, drugs and gFlHb (1.0 μM). (B) NADH oxidase activities of recombinant gFlHb (0.36 μM) in holo and FAD-less states and in the absence or presence of ABZ (150 μM). (C) Superoxide formation during the enzymatic activity of gFlHb (0.176 μM) and XO (0.176 μM) both in holo and FAD-less or XA-less states, in the presence and absence of ABZ (150 μM). (D) Detection of ABZSO by recombinant holo-gFlHb and FAD-less gFlHb (1.0 μM). (E) Detection of ABZSO by XO (1.0 μM) enzyme activity. Spectrum was determined at room temperature in 50 mM potassium phosphate buffer pH 7.5 in the presence (holoXO) or absence (XA-less XO) of 150 μM xanthine.

The unrelated rates of NADH consumption and ABZSO production prompted us to assess whether ABZSO could be produced from ABZ by gFlHb indirectly through a chemical mechanism, such as superoxide-mediated oxidation. For this, we carried out spectrophotometric assays to detect ABZSO in a semi-quantitative manner in enzyme assays that used either gFlHb holoenzyme or FAD- depleted enzyme. Due to the lower sensitivity of this technique compared to LC-MS/MS, it was necessary to standardize conditions where absorbance peaks of the product were not masked by other reaction components. It was first determined that ABZSO displays a characteristic peak at 288–293 nm. Based on this, an initial scan in the range 200–400 nm was run on the assay solution. Next ABZSO was added, and a new scan was run to confirm its presence as product of these reactions as shown in Fig 9. In these assays two different gFlHb concentrations (0.36 μM for NADH oxidase activity and 0.176 μM for ROS production) were used while excess ABZ was used at 150 μM. Fig 9A shows the absorbance peaks of reaction components and, of these, only NADH would cause a minimal interference on detection of ABZSO peaks while gFlHb, ABZ, DMF and FAD did not cause interference. This provides the option to withdraw FAD in reactions where gFlHb NADH oxidase activity had to be tested under enzyme inactivity (FAD- depleted). In general, recording a baseline for all components excepting ABZSO was done just before sample reading. Under these conditions, NADH consumption followed a similar kinetics either in the presence or absence of ABZ (Fig 9B). However, when the kinetics of ROS (i.e. O_2_^-^) production by active gFlHb was analyzed, a 160% increase within the reaction progress curve was observed in the presence of ABZ as compared to the rate in the absence of the drug (Fig 9C). As reference, xanthine oxidase (XO), another superoxide-producing enzyme, produced this species in a linear fashion over a 1-hr time period as expected; nevertheless, in this case superoxide production was decreased by 50% in the presence of ABZ (Fig 9C). No superoxide was detected with FAD-less gFlHb or XA-less XO (Fig 9C). The detection of a specific ABZSO peak under conditions favoring gFlHb activity was observed and mirrored the peak obtained after addition of ABZSO to the final reaction products whereas FAD-less gFlHb did not display any peak at 288–293 nm (Fig 9D). This demonstrated the oxidation of ABZ to ABZSO depended on superoxide production by gFlHb, suggesting an indirect chemical oxidation of ABZ by this enzyme. Furthermore, fully active xanthine oxidase also produced ABZSO from ABZ at a high rate and in a time-dependent manner (Fig 9E) confirming the involvement of O_2_^-^ in the oxidation of ABZ.

### Different mRNA levels of gFlHb are present in ABZ-resistant and ABZ-susceptible trophozoites

**Fig 10:**
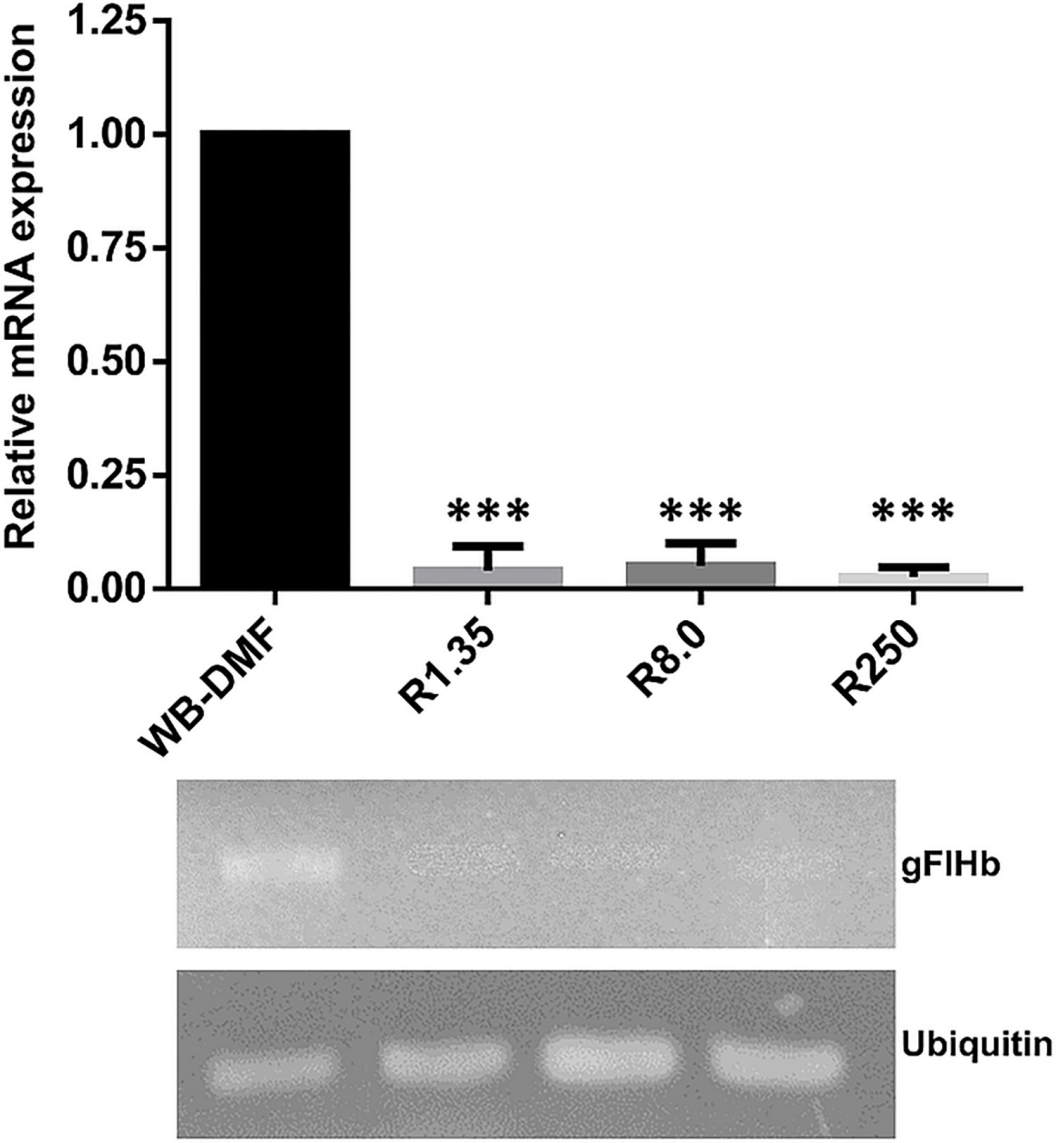
mRNA expression analysis of gFlHb of ABZ-resistant and ABZ–susceptible *G*. *duodenalis* trophozoites. The values represent the ratio of the optical densities of the PCR bands of gFlHb and internal standard ubiquitin. Densitometry values are the mean corresponding to n = 3 independent experiments. Asterisks denote a statistically significant difference (* p < 0.05, ** p < 0.01 and p < 0.001), as determined by ANOVA test.

To investigate the likely relation between gFlHb expression and adaptation to ABZ in resistant trophozoites, the mRNA levels of gFlHb were assessed in representative ABZ-resistant and ABZ-susceptible parasites. As seen in Fig 10A, the phenotype of all ABZ-resistant trophozoites confirmed those of previous studies. In ABZ-resistant culture lines, the mRNA levels of gFlHb were decreased or barely detected (Fig 10). These results suggest that a decrease in the expression of gFlHb is linked to a passive/pharmacokinetic resistance mechanism to ABZ in this parasite through a decreased production of cytotoxic metabolites as ABZSO.

### ABZ-resistant lines transfected with gFlHb become ABZ-susceptible upon increased expression of gFlHb

**Fig 11:**
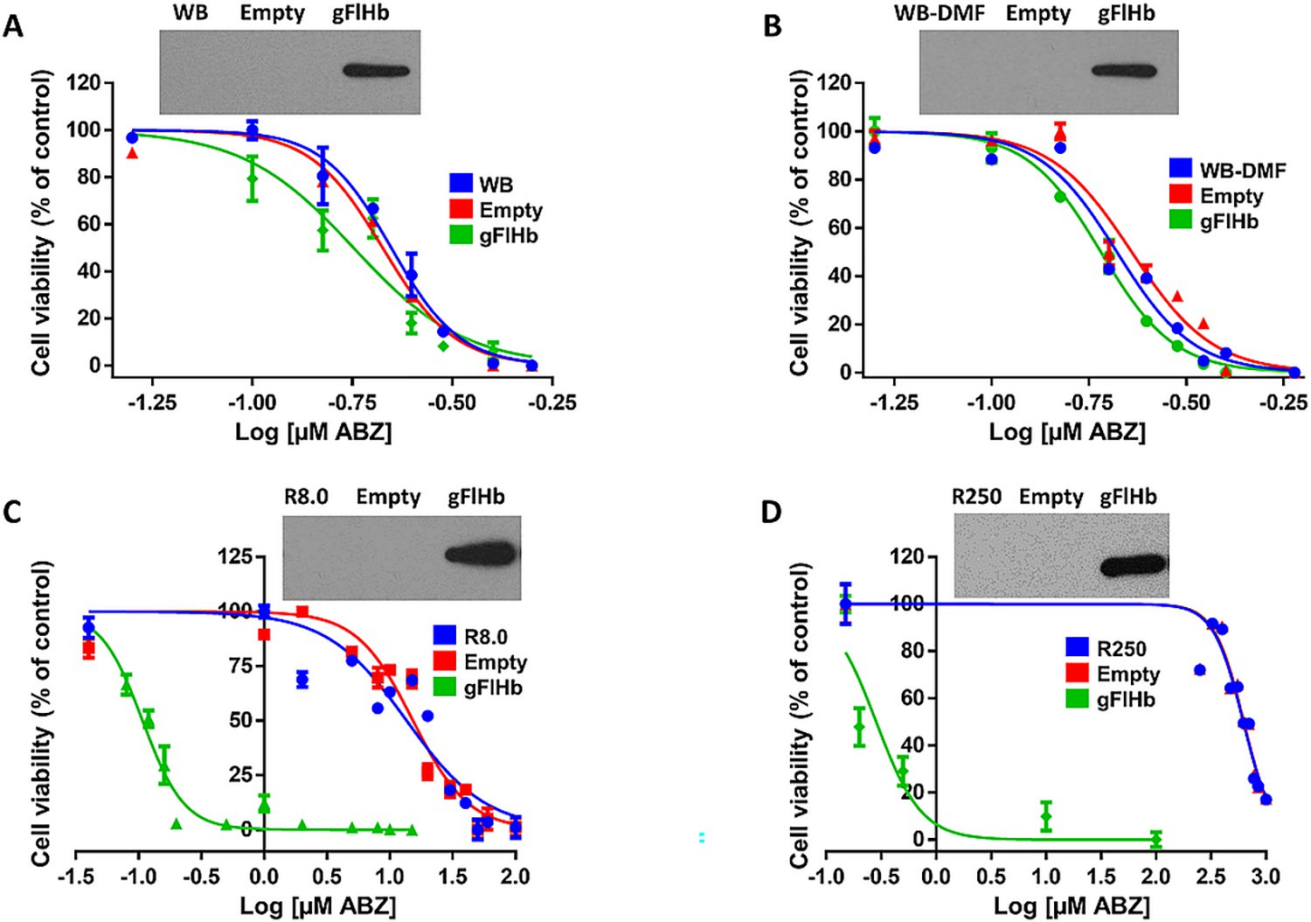
Concentration-viability curves of ABZ-susceptible and ABZ-resistant *G*. *duodenalis* trophozoites transfected with empty vector and pAC-gFlHb exposed to ABZ. (A) WB, (B)WB-DMF, (C) R8.0, and (D) R250 clones. Western blots indicating gFlHb overexpressed in trophozoites are shown in the upper part of the figure. Trophozoites were exposed to increasing ABZ concentrations (shown in logarithmic scale) for 24 h and cellular viability was determined by Trypan blue exclusion. The IC_50_ values correspond to n = 3 independent experiments. Standard deviations are shown for each ABZ concentration and for each IC_50_ determined.

In previous assays, it was shown that gFlHb catalyzes the oxidation of ABZ to ABZSO *in vitro*. We therefore proposed that the overexpression of gFlHb in ABZ-resistant and ABZ-susceptible trophozoites could alter their phenotypes. To test this proposal, functional assays were carried out in which the gFlHb gene was ligated into the expression vector *Giardia* pAC, which in turn was used to transfect ABZ-resistant and ABZ-susceptible trophozoite cultures. Expression of the exogenous, FLAG-tagged gFlHb protein was verified by Western blot assays using anti-FLAG antibodies (Fig 11, insets). The results obtained for WB trophozoites transfected with gFlHb showed a slight decrease in its IC_50_ (0.18 ± 0.04 μM) which was not significant with respect to the empty vector (0.21 ± 0.01) (Fig 11A). On the other hand, the WB trophozoites cultured in the presence of the vehicle (WB-DMF) and transfected to overexpress gFlHb decreased its IC_50_ by about 20% (0.19 ± 0.02 μM) when compared to the empty vector (0.23 ± 0.03 μM) (Fig 11B). The analysis of variance of the three independent transfection assays also showed that the WB-DMF trophozoites transfected with the empty vector had a statistically insignificant increase in IC_50_ with respect to the non-transfected control (0.21 ± 0.02), indicating that the vector has a negligible effect by itself on trophozoite survival. Remarkably, the ABZ-resistant trophozoite cultures R8.0 (Fig 11C) and R250 (Fig 11D) that were transfected with the gFlHb vector showed dramatic decreases of IC_50_ values in the presence of ABZ (80-fold and 2239-fold respectively), to values that were similar to those of the WB parental isolate (Table 3). These results are consistent with the lower mRNA expression of gFlHb in ABZ-resistant trophozoites and that the overexpression of gFlHb in ABZ-resistant trophozoites induces susceptibility to ABZ in these clones, and that ABZ-susceptible trophozoites become even more susceptible to this benzimidazole drug. These transfection experiments together with the *in silico* predictions and *in vitro* analyses using the purified enzyme ultimately support that gFlHb contributes, at least, to ABZ monooxygenation in *G*. *duodenalis*.

**Table 3:** Summary of IC_50_ values of ABZ in sensitive and resistant clones transfected with pAC and pAC_gFlHb.

Cell line	Non transfected	Empty vector	gFlHb-transfected	Fold change
WB	0.22 ± 0.02	0.21 ± 0.01	0.18 ± 0.04	≈1.2
WB-DMF	0.21 ± 0.02	0.23 ± 0.03	0.19 ± 0.02	≈1.2
R8.0	13.58 ± 3.38	15.25 ± 2.12	0.19 ± 0.01***	≈80
R250	622.2 ± 44.5	627.4 ± 44.1	0.28 ± 0.12***	≈2239

The IC_50_ values are given in μM. Values and errors (SE) shown are representative of n = 3 independent experiments. The asterisks indicate that values were different

* p< 0.05 and ***p<0.001, as determined by ANOVA test.

### ABZ-sensitive and ABZ-resistant lines transfected with gFlHb exhibit increased resistance to NO-generating compounds and MTZ

**Fig 12:**
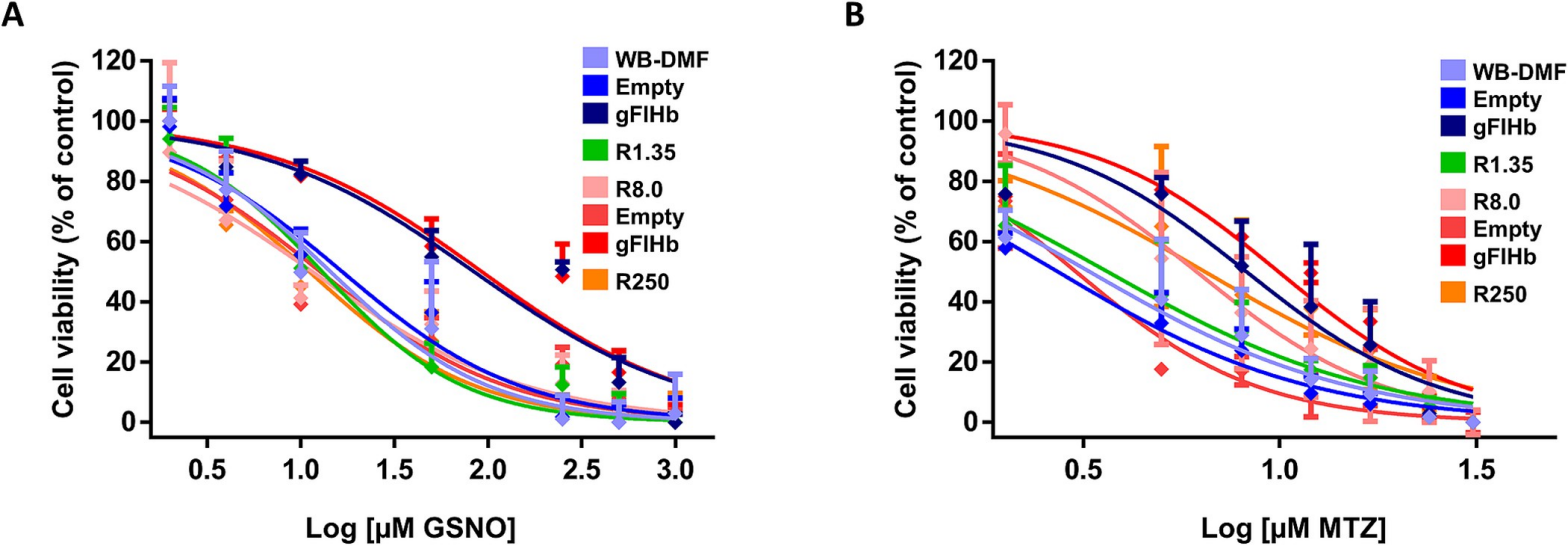
IC_50_ values of ABZ-sensitive and ABZ-resistant *G*. *duodenalis* trophozoites transfected and exposed to increasing MTZ (A) and GSNO (B) concentrations. Trophozoites were exposed to the indicated MTZ and GSNO concentrations for 24 h and cellular viability was determined by Trypan blue exclusion. The IC_50_ values correspond to n = 3 independent experiments. Standard deviations are shown for each drug concentration and for each IC_50_ determined.

As gFlHb provides protection from nitrosative stress by oxidizing nitric oxide (NO) to nitrate, the survival of trophozoites transfected with gFlHb upon exposure to NO could be affected. This was evaluated using the NO-generating compound nitric oxide S-nitrosoglutathione (GSNO). These assays showed that the ABZ-sensitive and ABZ-resistant trophozoites were sensitive to NO, and that the ABZ-resistant R1.35 (12 ± 2 μM) and R8.0 (10 ± 2 μM) and R250 (11 ± 1 μM) lines displayed a consistently greater sensitivity to GSNO as compared to WB-DMF (16 ± 5 μM), (Fig 12A) in good agreement with their respective levels of gFlHb expression. Likewise, transfection of gFlHb into WB-DMF and R8.0 trophozoites induced a lower sensitivity to NO which increased the IC_50_ by 5.3-fold (84 ± 4 μM) and 9.2-fold (93 ± 6 μM) compared to those of the empty vector (16 ± 0.8 and 11 ± 1 μM) respectively. Since other antigiardial compounds such as nitroheterocyclic drugs may cause nitrosative, the possibility of cross-resistance of gFlHb-transfected or non-transfected clones to MTZ was tested. These experiments showed a non-statistically significant but consistent increase of IC_50_ values in the ABZ-resistant (R1.35: 4 ± 1, R8.0: 5 ± 2 and R250: 7 ± 2 μM) in comparison to WB-DMF (2 ± 0.5 μM) trophozoites. The transfection of WB-DMF and R8.0 trophozoites with gFlHb showed a higher tolerance to MTZ as compared to their pairs with empty vector. WB and R8.0 clones increased by 2.8-fold (8 ± 1 μM) and 2.0-fold (10 ± 0.3 μM) in their IC_50_ compared to their empty vector-transfected pairs (3 ± 0.3 μM and 4 ± 1 μM respectively; Fig 12B). Taken together, these results show a role for gFlHb in response to nitrosative stress even in ABZ-resistant parasites, albeit at a lower extent for MTZ resistance, in agreement to distinct mechanisms working for MTZ resistance in *Giardia*.

## Discussion

In the absence of effective vaccines, the control of infections caused by parasitic protist and helminthic relies on chemotherapy. Although several kinds of drugs are available for the treatment of giardiasis, therapeutic failure occurs frequently and drug resistance is an issue of growing concern [64]⁠. Cases of drug-resistance in *Giardia* have been frequently reported [65]⁠. Mono- and multi-drug resistant cultures have been generated *in vitro*, including those resistant to both tinidazole and metronidazole [66]⁠ to furazolidone and quinacrine [67]⁠, to nitazoxanide and metronidazole [68]⁠, to albendazole and parbendazole, and to metronidazole and albendazole [24]. I⁠n particular, the generation and dissemination of ABZ-resistant *Giardia* may be caused by inadequate chemotherapy and use of suboptimal doses, either in the treatment of individual [69,70]⁠ or population via parasite control campaigns [19,71].⁠

Here, we show that gFlHb plays a role in the conversion of ABZ to ABZSO, based on the observations that: (1) ABZ stimulates NADH oxidation by gFlHb and ABZSO as a reaction product was detected and even quantified; (2) the addition of ABZ causes optical changes to the heme spectrum of gFlHb, suggesting a direct interaction; and (3) ABZ resistance in trophozoites is reversed when gFlHb is overexpressed in ABZ-resistant clones. The results present in this work provide clear evidence for a flavohemoglobin role in ABZ metabolizing in *G*. duodenalis and its participation in an antioxidant, pharmacokinetic mechanism of resistance to ABZ in this parasite.

Drug resistance in parasitic organisms has been extensively studied; however, the resistance response depends on the parasite, and the mechanism of action of each drug and its biotransformation. The resistance to ABZ in helminths and fungi has been correlated with the presence of mutations in the sequence of β-tubulin [21,22,72]⁠; likewise a mutation (E198K) has been reported in selected ABZ-resistant clones of *Giardia*, as well as chromosomal rearrangements [18,25,65]⁠. In other studies an increased expression of antioxidant enzymes [11,26]⁠ and cross-resistance to hydrogen peroxide [27]⁠ in ABZ-resistant clones has been demonstrated, indicating the importance of neutralizing free radicals in the resistant phenotype. On the other hand, the ability of this parasite to metabolize ABZ has been shown via the detection of its sulfoxide and sulfone metabolites using specific antibodies [73]⁠ and other studies have quantified ABZ metabolites by HPLC in ABZ-susceptible and ABZ-resistant clones; these assays, and other studies have quantified ABZ metabolites by HPLC in ABZ-susceptible and ABZ-resistant clones, in these studies, a lower accumulation of ABZSO and ABZSOO was observed in the ABZ-resistant clone R250 [11]⁠. These findings suggest that compared to ABZ-sensitive clones, the conversion of ABZ into ABZSO in ABZ-resistant clones is slower, or that the conversion of ABZSO into ABZSOO is faster. Furthermore, in ABZ-resistant clones, differential gene expression has been observed for some VSPs and other molecules involved in cytoskeletal function and/or antioxidant/energy metabolism [11,18,24,26,27]⁠. These reports suggest that resistance of *G*. *duodenalis* to ABZ is a multifactorial process, whereby different elements are involved in maintaining this phenotype, as reported for MTZ-resistant clones [74]. ⁠In addition, considering the plasticity in the biological responses of the parasite to cellular stress, the presence of multiple mutations in proteins targeted by ABZ is likely, besides the E198K change in the colchicine-binding domain of β-tubulin [25].⁠

In this study, we showed that ABZ was more potent than ABZSO and ABZSOO in drug-sensitive trophozoites, a feature consistent with other reports on the inhibition of tubulin polymerization [75]⁠. Although it has been suggested that ABZSOO lacks antiparasitic activity, we found that it displayed an effect over survival of trophozoites, at least in ABZ-sensitive clones (Fig 2). Another interesting finding was that ABZ-resistant trophozoites were also more tolerant to ABZSO and ABZSOO, an observation that emphasizes (a) the importance of *intracellular* generation of ABZSO and ABZSOO for the overall cytotoxicity of ABZ in *Giardia*; (b) the presence of multiple resistance mechanisms in this organism; and (c) the possibility of an altered ability of parasites to uptake *extracellular* ABZ and/or respective metabolites from the surroundings, potentially involving specific transporters, exchangers or channels (cf. [75]⁠) yet to be identified.

The addition of hemin to the clones studied here stimulated the mRNA expression of the gFlHb-encoding gene. Although hemin can be toxic to a variety of cell types [76–79]⁠ due to the production of ROS and protein ubiquitination [80–82]⁠. The addition of hemin at concentrations less than 50 μM had no effect on *Giardia* growth, albeit there was a positive relationship between extracellular hemin concentrations and parasite viability. The TYI-S-33 medium supplemented with 10% serum (HyClone) contains a low concentration of hemin (0.24 μM) that supports a good rate of trophozoite growth, and that at concentrations of 4 μM hemin even increases cell survival. In this context, the *Giardia* genome encodes five heme-containing proteins, namely four cytochrome *b*_5_ isoforms (gCYTB5 I-IV) and one flavohemoglobin (gFlHb) [83]⁠. Of these, gFlHb is the only hemoprotein with dioxygenase activity in this parasite [30,33]. F⁠urthermore, FlHbs have high affinities for imidazole and other azole inhibitors [34,57]⁠ and have a large hydrophobic and flexible active site [35]⁠. Consistent with these properties, *in silico* molecular docking suggested that gFlHb has favorable binding energies for ABZ and for certain CYP450 inhibitors. Flavohemoglobins are mainly known for their ability to neutralize nitrosative stress by acting as NOD in protists, fungi and bacteria [30,33]. S⁠ince FlHbs are absent from humans, the inhibition or subversion of gFlHb functions indicate that this enzyme may be an important target for anti-giardial chemotherapy.

To further study the functional role of gFlHb, this protein was cloned and expressed in *E*. *coli* strain BL21 (DE3) and the substrate specificity and catalytic properties of the recombinant enzyme characterized and the effect of its overexpression on the sensitivity to ABZ in transfected trophozoites explored. Initial analysis by molecular modeling showed that most of the conserved residues were in the active site of other family members, except two sequence inserts in the globin domain and FAD-binding domain [30]⁠ (Fig 4). These inserted sequences are absent from other FlHb family members, but tunnel analyses showed that these insertions are located in the same region of the enzyme and could be involved in gating substrate entry (S2 Fig). Furthermore, the structures of globins indicate the presence of internal cavities and tunnels [84]⁠ that may participate in multi-ligand catalysis. The comparison of *Giardia* gFlHb homologs in *E*. *coli* and *Alcaligenes eutropha* reveals additional small insertions and deletions (cf. [85]⁠), which could lead to subtle yet important differences in structural and domain orientation.

Regarding catalysis by flavohemoglobins, it is interesting that, although the protein folding is shared with other oxygen-binding globins, the specific structural features of the proximal side of the heme iron are typical of these peroxidases. Their proximal sides are characterized by strictly conserved residues: HisF8, TyrG5 and GluH23 (Fig 4C). The strong hydrogen-bond interactions between these residues increase the imidazolate character of HisF8, thereby stabilizing high-valent iron oxidation states and electron-poor iron-oxygen/nitrogen intermediates [62]⁠. The spectral properties of recombinant gFlHb resemble other flavohemoglobins; most importantly, the addition of NADH yields a Soret peak at 415 nm and to α- and β-bands at 548 and 580 nm that are characteristic of the Fe(II)-O_2_ adduct, suggesting that heme reduction occurs through FAD. These oxy-gFlHb values are consistent with previous reports on gFlHb (Soret 415 nm and α/β bands at 544 and 579 nm) [86]⁠, also observed in *E*. *coli* HMP (Soret 415 nm and α/β and bands 545 and 579 nm) [55]⁠. The interaction of oxy-gFlHb and ABZ produced wavelength shifts from 415 to 425 and only one band was detected at 555 nm, indicating that ABZ may bind to heme as an exogenous ligand and that this occurs at the distal site of the heme moiety. ABZSO and ABZSOO produced similar spectral changes, raising the possibility that these compounds could also be ligands of gFlHb. In addition, deoxy-gFlHb did not exhibit the spectroscopy changes upon addition of ABZ as the oxygen containing gFlHb did, contrary to what is expected for a dioxygenase-type enzyme interacting with its substrate. This information suggests that the oxygen atom is not only needed for catalysis by gFlHb but that its presence optimizes gFlHb-ABZ interactions. The spectroscopic changes described for oxy-gFlHb have been previously observed in the active site of FlHb from *Ralstonia eutropha*, *Saccharomyces cerevisiae*, *Staphylococcus aureus* and, *Alcaligenes eutropha* interacting with azole inhibitors such as miconazole, econazole and ketoconazole [34,55–57]⁠. Interestingly, ABZ and ABZSO did not inhibit the NADH oxidase activity of gFlHb, but rather stimulated it by as much as 300%; however, this stimulation decreased when substrate concentration is increased, which suggest a saturated or transitory enhancing effect. In addition, although ABZSOO interacts with the active site, it does not modify the specific activity of gFlHb, which is consistent with the fact that sulfone is the last product of the biotransformation of ABZ. ABZ does not bind covalently to gFlHb as the azole inhibitors do and the mechanical movement induces separation of the enzyme-substrate complex. These data suggest a direct interaction of ABZ and its metabolites with the catalytic site of gFlHb without inhibiting its NADH oxidase activity (turnover: 14.5 nmol NADH•nmol gFlHb^-1^•min^-1^); furthermore, the LC-MS/MS data clearly indicate that gFlHb produces ABZSO from ABZ in the presence of oxygen and NADH, but at a very slow rate (turnover: 0.01 nmol ABZ•nmol gFlHb^-1^•min^-1^). Therefore, we endeavored to explain the quantitatively unrelated activities between NADH consumption and ABZSO production. We infer that gFlHb, as a member of NOD family, has a multi-step catalytic cycle for the oxidation of nitric oxide to nitrate involving the initial oxidation of NADH to promote conversion of FAD into FADH_2_ (step 1), which, in turn, transfers electrons to heme-bound iron (steps 2 and 3), which, upon reduction, allows O_2_ binding that, upon iron reoxidation, produces O_2_^-^ (step 4) that finally oxidizes NO to form NO_3_^-^ (step 5) (cf. [87]⁠).

Regarding the first step, if ABZ were a *bona fide* substrate of gFlHb, our spectroscopic and LC-MS/MS data shown in Figs 6A, 7, and 8 would not adequately support this notion, although direct interactions between the enzyme and the drug do exist (Fig 6B). This latter statement was further supported when experiments were carried out under enzyme excess, where NADH oxidase activity was enhanced by 20–25 nM ABZ but not at higher drug concentrations. This superoxide observation could be explained by the presence of a high affinity site in gFlHb (at allosteric moieties or at active site), where a modulating effect on NADH oxidase activity is saturated and/or transitory. Thus, the observed peak of NADH oxidase activity could correspond to the enzyme subpopulation, effectively stimulated by existing ABZ. The other step in gFlHb catalysis likely modulated by ABZ was the higher production of O_2_^-^ (Fig 9C). As imidazoles bind with high affinity to the active site, it is conceivable that ABZ could bind via its imidazole moiety to gFlHb active site to stimulate ROS production like a typical FlHb inhibitors [50,88]⁠. It is also tempting to propose that the interaction of ABZ with gFlHb (in an as yet undefined site) might modulate NADH oxidase activity and this phenomenon could resemble the cooperative and allosteric mechanism of O_2_ and NO that is mediated by the formation of tunnels that allows active movement in the active site [89]⁠.

The simultaneous determination of NADH oxidase activity, O_2_^-^ production and ABZSO detection in gFlHb holoenzyme reactions by spectroscopy shed light on the mechanism involved in ABZ→ABZSO conversion. Under excess, ABZ over gFlHb (where a typical enzyme catalysis would occur), it was observed that ABZ is oxidized by superoxide generated through the NADH oxidase activity of gFlHb, moreover XO xanthine oxidase, an enzyme that produces superoxide during its catalytic cycle, was also able to produce ABZSO. These observations highlight the role of the superoxide in ABZ metabolism in *G*. *duodenalis* through a mechanism that does not involve direct interaction between the enzyme and ABZ. These observations provide not only insights into the metabolism of (alkyl)sulfur-bearing benzimidazole xenobiotics of clinical use, such as ABZ and fenbendazole, but raise the possibility that other organisms harboring superoxide-producing enzymes are able to metabolize ABZ in this way. This, in turn, is consistent with the observation that gFlHb, like other flavohemoglobins, is a multi-substrate enzyme [34,90]⁠.

The slow production of ABZSO by gFlHb (step 5 of catalysis) could not be fully explained. We still propose that a catalytic mechanism might take place in the oxygenation step involving an enzymatic partner as exists for other heme-containing enzymes capable of metabolize ABZ [91]⁠ (which includes several CYP450 isoforms (e.g. C3A4, CYP2J2, CYP2C19) [10] and other NODs).

The expression of flavohemoglobin is usually related to oxidative and nitrosative stresses, and is considered as a molecular oxygen defense against nitric oxide donors and other drugs [92–95]⁠. Consistent with these observations, gFlHb is overexpressed in trophozoites exposed to nitric oxide donors, such as NONOate and GSNO [33]⁠. Although ABZ produces oxidative stress by induction of ROS [27]⁠, the levels of gFlHb expression are lower or negligible in ABZ-resistant lines (Fig 10). Based on this observation, we characterized the phenotype of ABZ-susceptible and ABZ-resistant clones when gFlHb is constitutively overexpressed by transfection of trophozoites with the gFlHb gene that was ligated into the expression vector *Giardia* pAC. Overexpression of gFlHb had minimal effects on ABZ-susceptible clones to this drug, but significant effects on ABZ-resistant clones, with the IC_50_ towards ABZ decreasing 80-fold and 2239-fold in R8.0 and R250 clones, respectively, approaching the IC_50_ values of the ABZ-susceptible clones. These results suggest participation of a *passive* resistance mechanism similar to what is observed for ferredoxins in MTZ resistance [74]. Thus, a lower expression level of enzymes involved in ABZ biotransformation, in this case gFlHb, significantly may diminish the production of cytotoxic drug metabolites inside the cell. Although the presence of *active* mechanisms of resistance to ABZ has been suggested by the increased expression of proteins involved in antioxidant response in resistant clones [11,26]⁠, the data of the present work suggest that gFlHb plays a passive/pharmacokinetic role in acquired ABZ-resistance of *Giardia* by limiting the production of the cytotoxic metabolite ABZSO, which is in agreement with previous quantitative studies [11]⁠.

In conclusion, in the present study, we have discovered that the NO-scavenging enzyme flavohemoglobin of *G*. *duodenalis* is a multifunctional protein which is likely responsible for the partial biotransformation of ABZ into ABZSO, and that the downregulation of its expression/transcription is linked to an ABZ-resistant phenotype. This enzyme catalyzes at least, the sulfoxidation of ABZ to its metabolite ABZSO, constituting a novel system for ABZ biotransformation. The role of gFlHb in the resistance of *Giardia* to ABZ reveals that this enzyme acts in a passive/pharmacokinetic-mediated drug resistance mechanism. These results contribute to our understanding of the molecular mechanisms of ABZ resistance in *G*. *duodenalis* to ABZ and provide a platform for drug design or drug repurposing to find alternative treatments of giardiasis that target this biotransformation system.

## Supporting information

S1 Fig Validation plots for the protein structure model of *G*. *duodenalis* flavohemoglobin. (A) Ramachandran plot summary of gFlHb model using Procheck showing 71.0% residues in favorable, 21.2% residues in additional allowed, 5.9% in generously allowed and 2.0% in disallowed regions. (B) Validation of gFlHb model using ProSa-Web shows Z-score as -7.37. Veryf 90.17% and (C) Normalized QMEAN4 plot shows the standard deviation of the model.
(TIF)

S2 Fig Intramolecular tunnels inferred in the protein model of gFlHb. Tunnel connecting the heme distal site to protein surface. The tunnel is displayed in magenta, the insertion of sequences of gFlHb in the globin and FAD domains are highlighted in red and blue, respectively. The dotted box represents the active site, and the starting point is depicted as a yellow star. The accessible path was identified by CANVER 3.0.3.
(TIF)

S3 Fig (A) Spectral characteristics of reagents and drugs. (B) Effects of reagents and drugs in spectral characteristics of recombinant oxidized gFlHb (6.6 μM). The experiments were performed at 37°C in 50 mM potassium phosphate buffer (pH 7.5).
(TIF)

S4 Fig The docked poses and molecular interactions of drugs in the active site of gFlHb. The docked poses of (A) Econazole (RMSD = 2.8 Å), (B) Ketoconazole (RMSD = 3.2 Å), (C) Miconazole (RMSD = 2.8 Å) with residues of the catalytic site. (D) Predicted affinities for of these drugs towards gFlHb as determined using AutoDock/Vina and SwissDock.
(TIF)

S5 Fig NADH oxidase activity of recombinant gFlHb. The reaction was performed in 50 mM potassium phosphate buffer to which 150 μM NADH was added at time zero; later 0.37 μM gFlHb was added to reach a baseline and finally 1 μM FAD was added to initiate the reaction. A) NADH oxidase activities of recombinant gFlHb in the presence of different ABZ concentrations. B) NADH oxidase activities of gFlHb with SOD (5 U) and CAT (13U) in the presence of different ABZ concentrations. C) Relative specific activity of gFlHb in complex with ABZ. Values and errors (SE) shown are representative of n = 3 independent experiments (D). Specific activity and percentage of gFlHb in complex with ABZ. The specific activity units are *μmol/min*mg protein
(TIF)

S6 Fig Superoxide formation in the gFlHb enzymatic assay. It was determined that 5U SOD completely abolished superoxide accumulation in the reaction mixture.
(TIF)
