# Supplementary figures and images for: Giardia duodenalis: Flavohemoglobin is involved in drug biotransformation and resistance to albendazole
Source: PLoS Pathog. 2022 Sep 27;18(9):e1010840. doi: 10.1371/journal.ppat.1010840 (PMC9514659; doi:10.1371/journal.ppat.1010840)

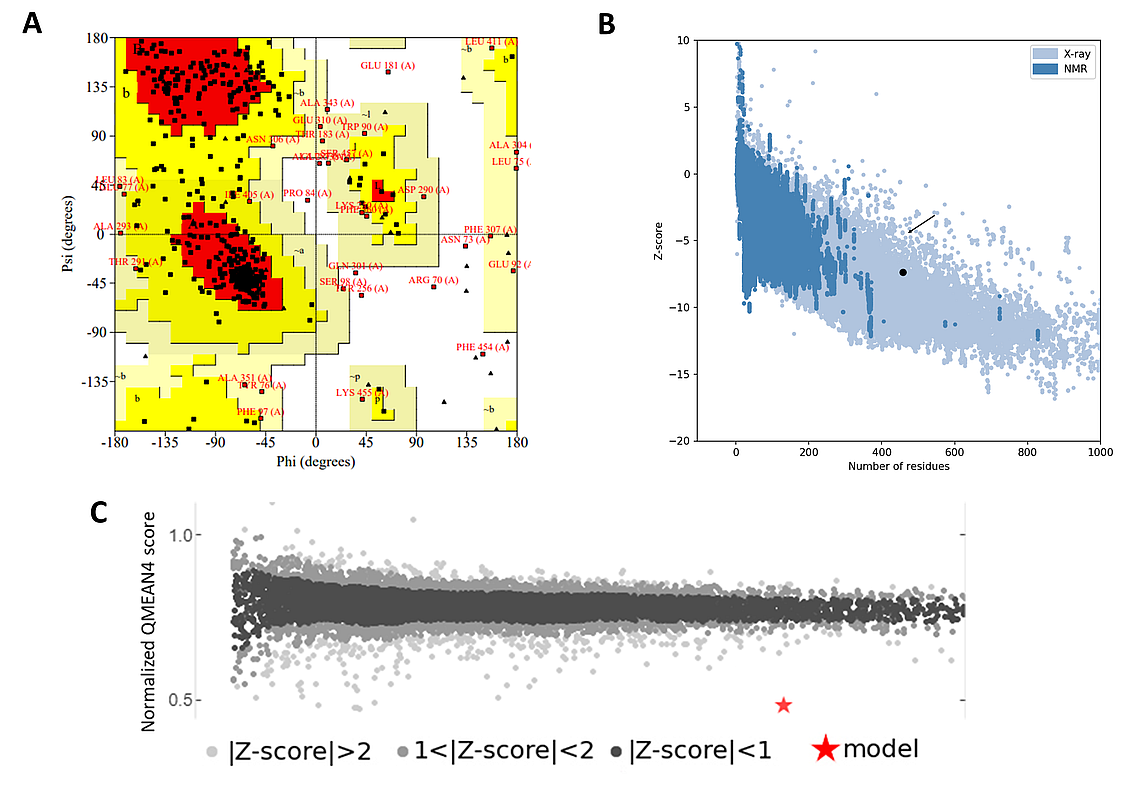

Supplement: S1 Fig — (A) Ramachandran plot summary of gFlHb model using Procheck showing 71.0% residues in favorable, 21.2% residues in additional allowed, 5.9% in generously allowed and 2.0% in disallowed regions. (B) Validation of gFlHb model using ProSa-Web shows Z-score as -7.37. Veryf 90.17% and (C) Normalized QMEAN4 plot shows the standard deviation of the model. (TIF) [file ppat.1010840.s001.TIF]

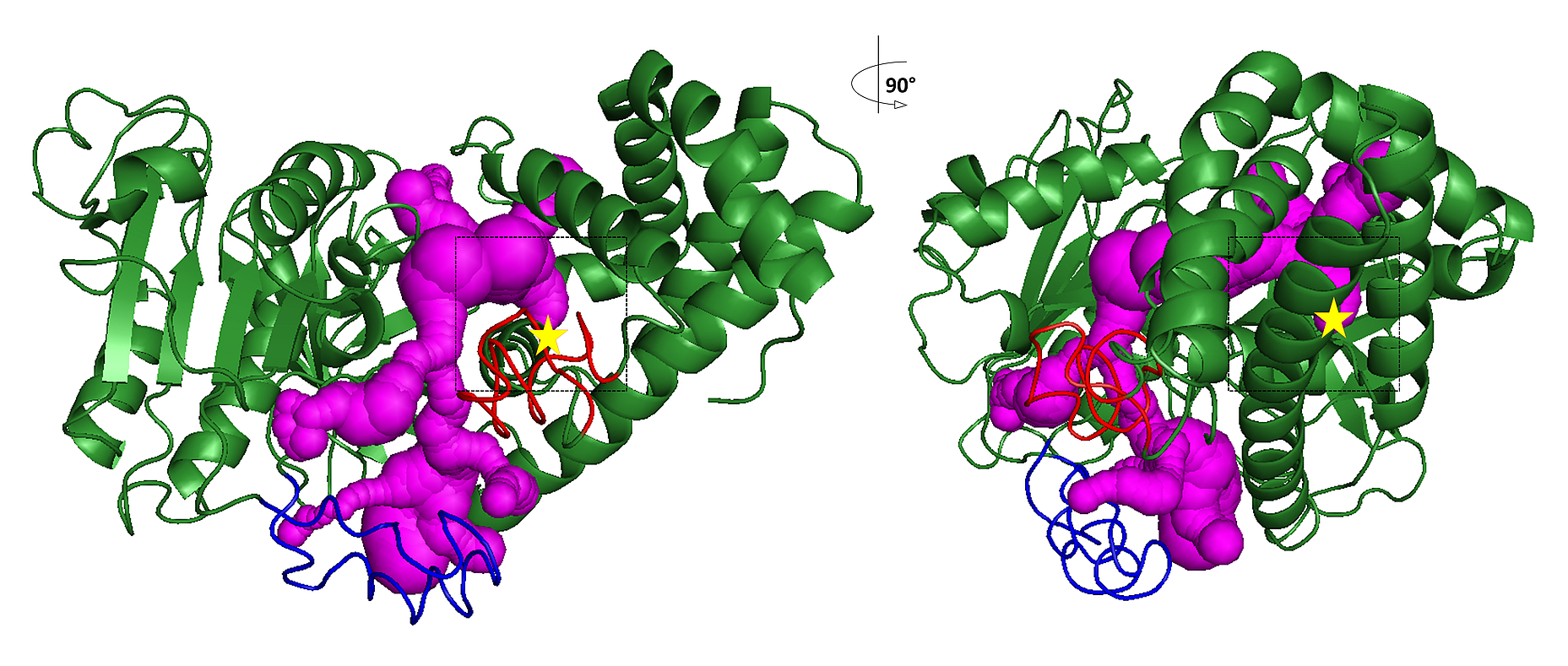

Supplement: S2 Fig — Tunnel connecting the heme distal site to protein surface. The tunnel is displayed in magenta, the insertion of sequences of gFlHb in the globin and FAD domains are highlighted in red and blue, respectively. The dotted box represents the active site, and the starting point is depicted as a yellow star. The accessible path was identified by CANVER 3.0.3. (TIF) [file ppat.1010840.s002.TIF]

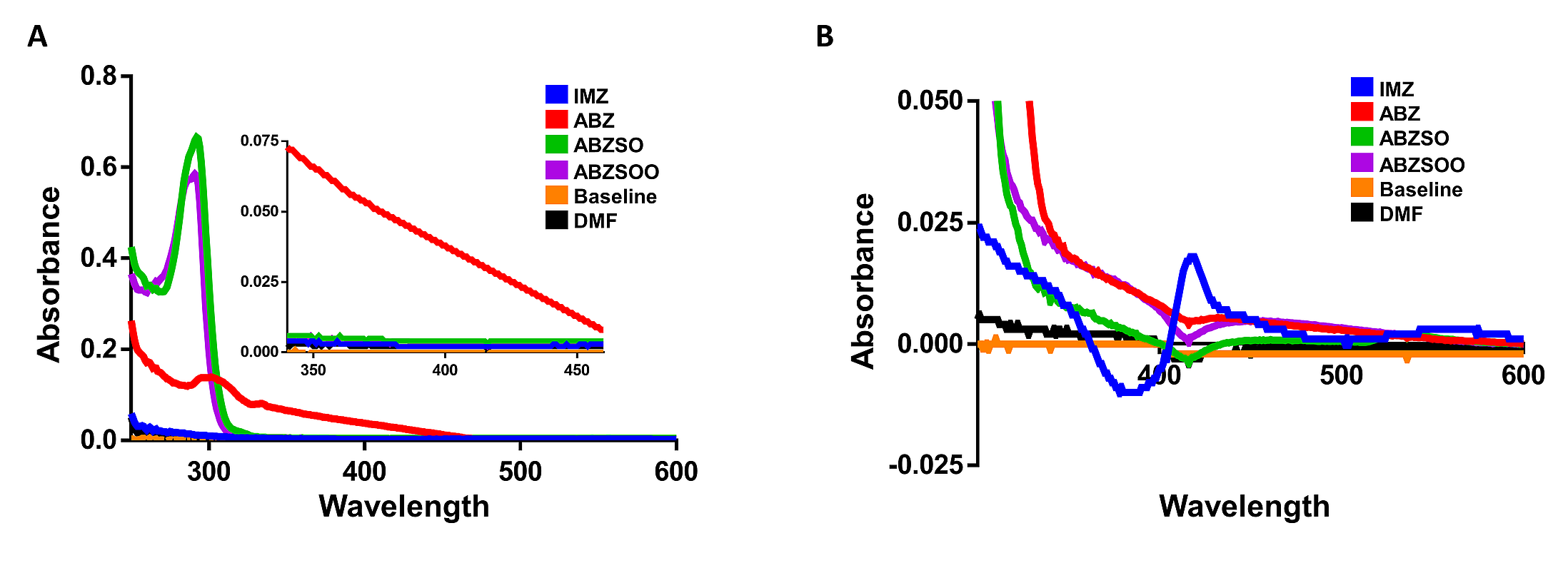

Supplement: S3 Fig — (A) Spectral characteristics of reagents and drugs. (B) Effects of reagents and drugs in spectral characteristics of recombinant oxidized gFlHb (6.6 μM). The experiments were performed at 37°C in 50 mM potassium phosphate buffer (pH 7.5). (TIF) [file ppat.1010840.s003.TIF]

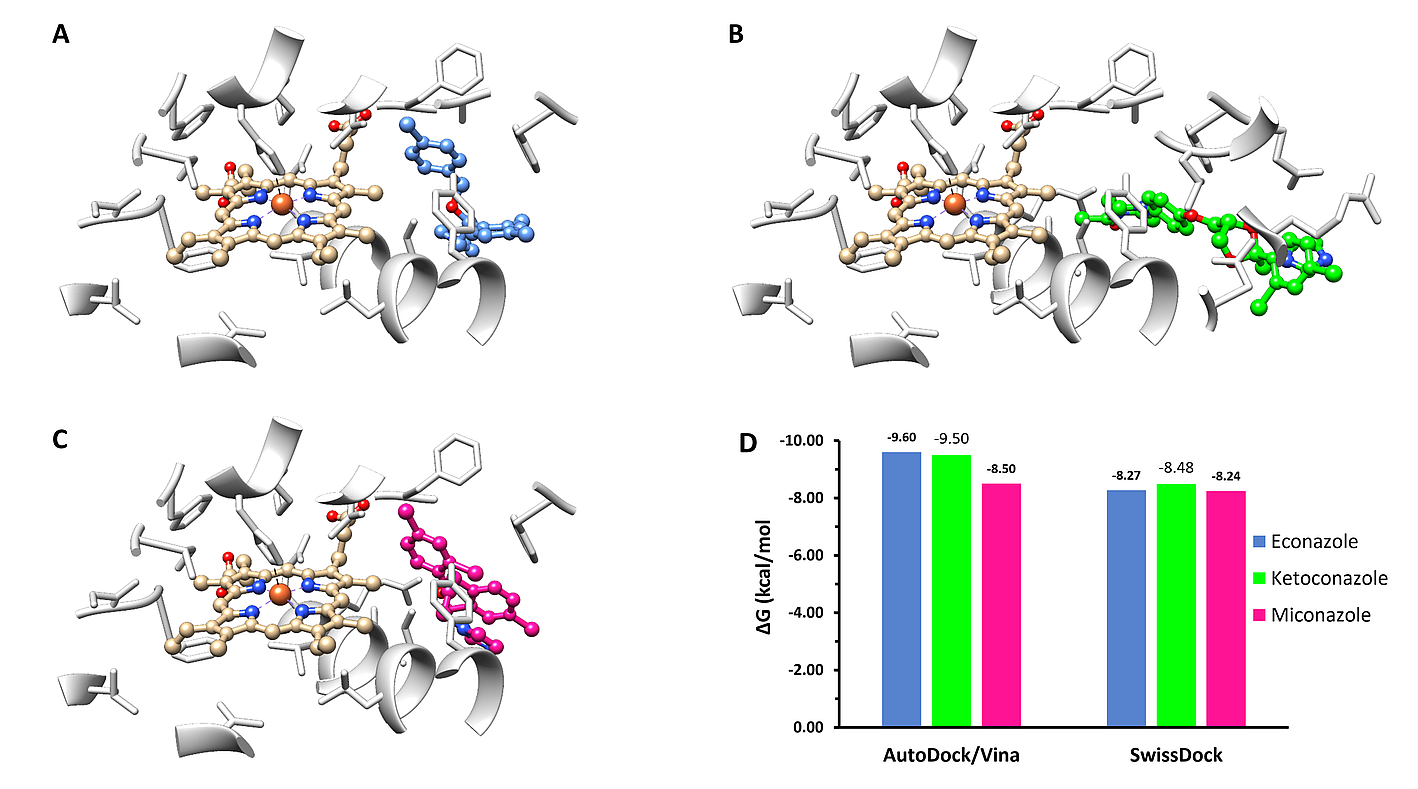

Supplement: S4 Fig — The docked poses of (A) Econazole (RMSD = 2.8 Å), (B) Ketoconazole (RMSD = 3.2 Å), (C) Miconazole (RMSD = 2.8 Å) with residues of the catalytic site. (D) Predicted affinities for of these drugs towards gFlHb as determined using AutoDock/Vina and SwissDock. (TIF) [file ppat.1010840.s004.tif]

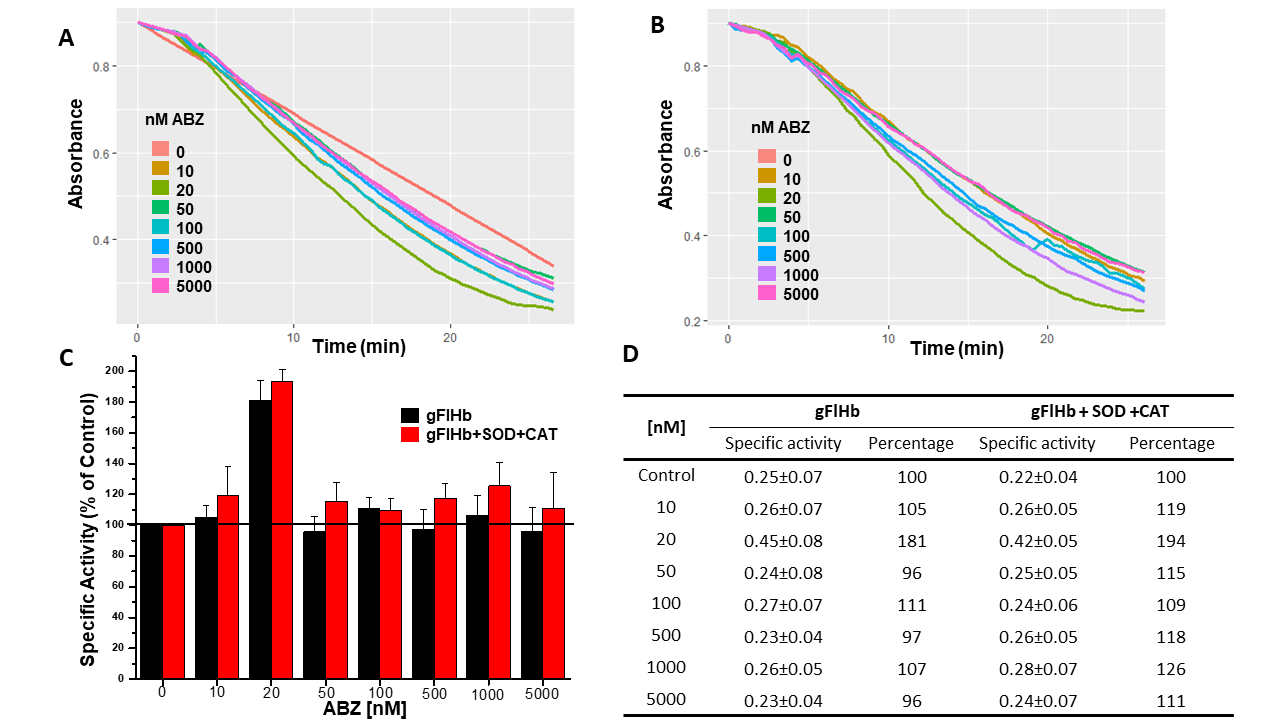

Supplement: S5 Fig — The reaction was performed in 50 mM potassium phosphate buffer to which 150 μM NADH was added at time zero; later 0.37 μM gFlHb was added to reach a baseline and finally 1 μM FAD was added to initiate the reaction. A) NADH oxidase activities of recombinant gFlHb in the presence of different ABZ concentrations. B) NADH oxidase activities of gFlHb with SOD (5 U) and CAT (13U) in the presence of different ABZ concentrations. C) Relative specific activity of gFlHb in complex with ABZ. Values and errors (SE) shown are representative of n = 3 independent experiments (D). Specific activity and percentage of gFlHb in complex with ABZ. The specific activity units are *μmol/min*mg protein (TIF) [file ppat.1010840.s005.tif]

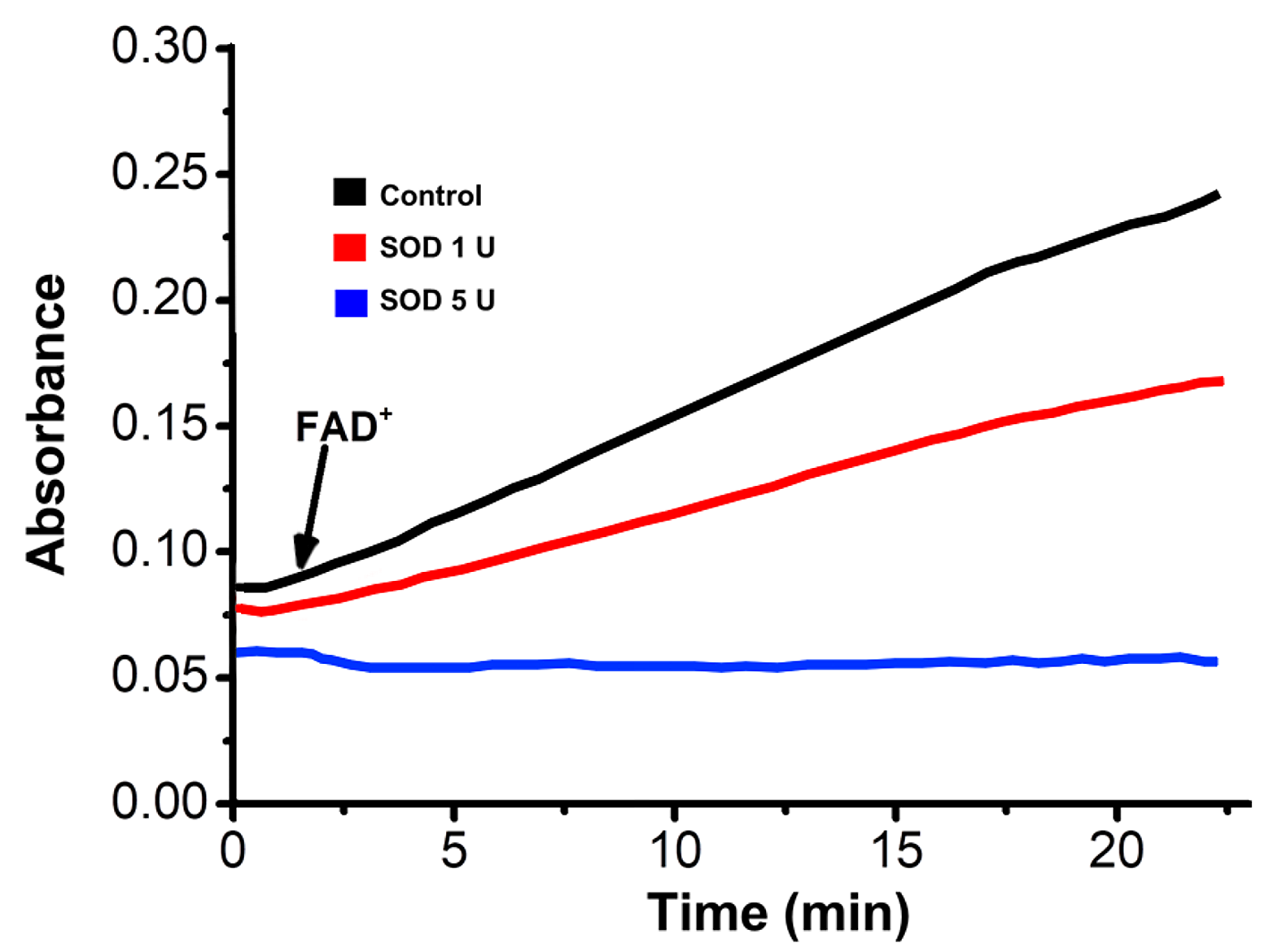

Supplement: S6 Fig — It was determined that 5U SOD completely abolished superoxide accumulation in the reaction mixture. (TIF) [file ppat.1010840.s006.TIF]
